# The RegA regulon exhibits variability in response to altered growth conditions and differs markedly between *Rhodobacter* species

**DOI:** 10.1099/mgen.0.000081

**Published:** 2016-10-21

**Authors:** Heidi S. Schindel, Carl E. Bauer

**Affiliations:** Biochemistry, Indiana University Bloomington, Simon Hall MSB, 212 S. Hawthorne Dr., Bloomington, IN 47405-7003, USA

**Keywords:** redox regulation, global transcription factor binding, PrrA homologue, microbial photosynthesis

## Abstract

The RegB/RegA two-component system from *Rhodobacter capsulatus* regulates global changes in gene expression in response to alterations in oxygen levels. Studies have shown that RegB/RegA controls many energy-generating and energy-utilizing systems such as photosynthesis, nitrogen fixation, carbon fixation, hydrogen utilization, respiration, electron transport and denitrification. In this report, we utilized RNA-seq and ChIP-seq to analyse the breadth of genes indirectly and directly regulated by RegA. A comparison of mRNA transcript levels in wild type cells relative to a RegA deletion strain shows that there are 257 differentially expressed genes under photosynthetic defined minimal growth medium conditions and 591 differentially expressed genes when grown photosynthetically in a complex rich medium. ChIP-seq analysis also identified 61 unique RegA binding sites with a well-conserved recognition sequence, 33 of which exhibit changes in neighbouring gene expression. These transcriptome results define new members of the RegA regulon including genes involved in iron transport and motility. These results also reveal that the set of genes that are regulated by RegA are growth medium specific. Similar analyses under dark aerobic conditions where RegA is thought not to be phosphorylated by RegB reveal 40 genes that are differentially expressed in minimal medium and 20 in rich medium. Finally, a comparison of the *R. capsulatus* RegA regulon with the orthologous PrrA regulon in *Rhodobacter sphaeroides* shows that the number of photosystem genes regulated by RegA and PrrA are similar but that the identity of genes regulated by RegA and PrrA beyond those involved in photosynthesis are quite distinct.

## Data Summary

Raw sequence data from these RNA-seq and ChIP-seq studies can be accessed via the NCBI Sequence Read Archive with the accession number SRP076177 (http://trace.ncbi.nlm.nih.gov/Traces/sra/?study=SRP076177).

## Impact Statement

The RegA regulon from *Rhodobacter capsulatus* is determined to be quite large with direct and indirect control of genes involved in energy generation and energy utilization that ultimately affects the redox state of the ubiquinone pool. Directly and indirectly regulated targets include genes involved in central metabolism, photosynthesis, respiration, motility and iron transport. RegA is not thought to be phosphorylated by its cognate histidine kinase RegB under aerobic conditions, yet there are a subset of genes regulated by RegA aerobically. Finally, the *R. capsulatus* RegA regulon was also compared to the PrrA regulon from *Rhodobacter sphaeroides*. RegA and PrrA are orthologues exhibiting 87 % sequence similarity, with RegA known to functionally complement PrrA. Surprisingly, both species only directly control photosystem gene expression in common with considerable divergence in function beyond photosynthesis. Similar divergence in target genes was also recently reported for Fnr orthologues in these two *Rhodobacter* species. Consequently, it is not possible to extrapolate similar function between highly homologous global regulatory proteins in related species without additional bioinformatics or experimental analyses.

## Introduction

The survival of bacteria in a variety of different environments requires that they constantly assess and adapt to their surroundings. Assessment of environmental changes often involves two-component systems that alter gene expression that in turn alters cellular physiology (Bijlsma & Groisman, 2003; Capra & Laub, 2012). One of the many signals that two-component systems sense is the presence or absence of environmental oxygen, which is critical for regulating a switch between aerobic respiratory and anaerobic growth modes (Wu *et al.*, 2012). This is particularly true for metabolically diverse anoxygenic purple photosynthetic bacteria that engage in dark aerobic respiration in the presence of oxygen or photosynthetic growth under light anaerobic conditions (Bauer *et al.*, 2009).

Many alphaproteobacteria sense changes in oxygen tension using the RegB/RegA two-component system (Elsen *et al.*, 2004; Wu & Bauer, 2008). RegB and RegA were discovered during a genetic screen for factors required for anaerobic synthesis of the *Rhodobacter capsulatus* photosystem (Sganga & Bauer, 1992; Mosley *et al.*, 1994). Sequence and biochemical analysis indicated that RegB and RegA comprise a cognate histidine kinase–response regulator pair, respectively, where RegB autophosphorylates itself under reducing conditions (symptomatic of low oxygen tension) and subsequently transfers a phosphate to RegA (Mosley *et al.*, 1994; Inoue *et al.*, 1995; Bird *et al.*, 1999). Once phosphorylated, RegA binds DNA to alter gene expression and, ultimately, cellular metabolism (Du *et al.*, 1998; Bird *et al.*, 1999; Hemschemeier *et al.*, 2000).

RegB is a membrane-spanning histidine kinase that senses changes in redox through multiple mechanisms. The cytoplasmic portion of RegB contains a redox-reactive cysteine that can be oxidized by molecular oxygen to a disulfide bond or to a sulfenic acid, both of which inhibit RegB autophosphorylation (Swem *et al.*, 2003; Wu *et al.*, 2013). RegB also senses changes in cellular redox by a direct interaction with electron-carrying ubquinones (Swem *et al.*, 2006; Wu & Bauer, 2010). Full-length RegB weakly binds to both oxidized and reduced ubiquinone with only oxidized ubiquinone inhibiting kinase activity ~6-fold (Wu & Bauer, 2010). It has been proposed that the low binding affinity to ubiquinone allows it to frequently exchange bound ubiquinone with the large ubiquinone pool in the membrane (Wu & Bauer, 2010). This would allow RegB to sample the overall redox state of the ubiquinone pool that fluctuates in response to oxygen availability (Grammel & Ghosh, 2008).

In addition to activating anaerobic expression of the photosystem (Du *et al.*, 1998; Hemschemeier *et al.*, 2000; Willett *et al.*, 2007), biochemical analyses showed that phosphorylated RegA binds to promoter regions that express respiratory cytochromes *cbb_3_* oxidase and ubiquinol oxidase and the electron shuttling cytochromes *bc_1_*, *c_2_* and *c_y_*, several of which are shared by photosynthesis and respiration (Du *et al.*, 1998; Swem *et al.*, 2001). Beyond photosystem and respiration, RegB/RegA also controls carbon fixation, nitrogen fixation, hydrogen utilization and denitrification (Elsen *et al.*, 2000; Vichivanives *et al.*, 2000; Swem *et al.*, 2001). Surprisingly, studies also show that RegA alters expression of genes in the presence of oxygen, which is a growth condition that should inactivate the kinase activity of RegB (Elsen *et al.*, 2000; Swem *et al.*, 2001; Swem & Bauer, 2002).

Previous studies have utilized biochemical techniques such as DNaseI footprinting, electrophoretic mobility shift assays, *lacZ* fusion expression assays and quantitative reverse transcriptase PCR (qRT-PCR) to determine genes under the control of RegA. However, these techniques require a predetermined target for analysis. In contrast, a global approach using genome sequencing-based RNA-seq does not require a target to be chosen beforehand and can therefore reveal new previously unexpected regulatory targets (Croucher & Thomson, 2010; Sorek & Cossart, 2010). In this study, we performed RNA-seq experiments under both aerobic and anaerobic (photosynthetic) conditions with mRNA from wild-type (WT) and *ΔregA* *R. capsulatus* cells to determine the changes in gene expression that occur through the RegB–RegA signalling cascade. We also determined which set of regulated genes are under direct control of RegA by performing chromatin immunoprecipitation of ectopically tagged RegA that was crosslinked to DNA *in vivo* with formaldehyde followed by deep sequence analysis of captured DNA segments (ChIP-seq) (Park, 2009). Under photosynthetic conditions, members of the RegB–RegA regulon vary depending on whether the cells are grown in defined minimal medium or in complex rich medium, indicating that RegA probably interacts with additional transcription factors to regulate different genes under different nutrient conditions. A similar pattern of medium dependence was observed in the extent of the RegA regulon under aerobic conditions with the caveat that significantly fewer genes are regulated by RegA under aerobic conditions than observed anaerobically. When compared to a recent RNA-seq and ChIP-seq study on the orthologous PrrA system from *Rhodobacter sphaeroides* (Imam *et al.*, 2014), the total number of regulated genes is similar, but the identity of genes regulated by these two photosynthetic species outside of photosystem genes is surprisingly quite different.

## Methods

### Strains and growth conditions.

The wild-type *R. capsulatus* strain (SB1003) has been described previously (Yen & Marrs, 1976). The *ΔregA* strain (DS05) was constructed by PCR amplification of 550 bp regions flanking the *regA* gene using primers regAdelXbaF (ATTCTAGAAAACCGAGCCTTGTC), regAdelXhoR (ATCTCGAGGAATTCTTCTTCGGC), regAdelXhoF (ATCTCGAGAGCCCGCGATAAACA) and regAdelSacR (ATGAGCTCGAGGATCTGAAACTC). The two fragments were cleaved with *Xba*I/*Xho*I and *Xho*I/*Sac*I, respectively, and ligated into pZJD29a cleaved with *Xba*I and *Sac*I. The resulting plasmid was mated into *R. capsulatus* using S17-1 λpir. Single recombinants were selected by gentamicin resistance and double rectype calcium-bindinombinants were selected on peptone-yeast extract (PY) plates containing 4 % sucrose.

Cells were grown in PY medium or in RCV with malate as the sole carbon source (Weaver *et al.*, 1975). Aerobically and photosynthetically grown cells were harvested when they reached an OD_660_ of 0.3. For RNA-seq sample collection, cell cultures were chilled with an ice water bath from which 750 µl of cell culture was extracted, centrifuged at 6000 r.p.m. for 10 min, followed by freezing at −80 °C until further processing.

### RNA-seq.

Cell pellets were resuspended in 100 µl of TE (10 mM Tris-HCl, 1 mM EDTA, pH 8) buffer containing 10 mg lysozyme ml^−1^ and incubated for 3 min at room temperature. Total RNA was isolated using the Bioline Isolate II RNA extraction kit. Following RNA extraction, DNA was removed by incubation with 1 unit of Turbo DNAse for 30 min at 37 °C followed by cleaning the RNA samples with a Qiagen MinElute RNA Clean-up kit. rRNA was then depleted and libraries were created using a Scriptseq Complete Kit for bacteria. Sequencing was completed on an Illumina Nextseq using the High Output 75 cycle. Data were trimmed using Trimmomatic with a sliding window of 5 : 25 with a minimum read length of 40 bp. Trimmed reads were aligned to the *R. capsulatus* chromosome with Bowtie2 (Langmead & Salzberg, 2012), followed by HTSeq-count (Anders *et al.*, 2015) to quantify read numbers. Raw counts generated from the HTSeq-count program were used to generate differentially expressed genes with the DESeq2 package in R (Robles *et al.*, 2012; Love *et al.*, 2014).

### RegA ChIP constructs.

All ChIP-seq experiments utilized tagged RegA on a plasmid in a *ΔregA* background. The WT 3×-FLAG RegA construct was created by amplifying the SenC-RegA promoter and coding region using primers GAACTCGAGTTGCCGACATGTCGAATTCCG (forward) and GAAACTAGTTTATCGCGGGCTGCGTTTGGC (reverse). The PCR product was digested with *Xho*I and *Spe*I and cloned into plasmid pBBR1MCS5 (Kovach *et al.*, 1995). The RegA gene was then amplified with primer CC**ATGGACTACAAAGACCATGACGGTGATTATAAAGATCAT GACATCGACTACAAG**GATGACGATGACAAGGCTGGCTCCGCTGCTGGTTCTGGCGCCGAAGAAGAATTCGCCGCGAACTCGGAACTCGG (forward) and primer GAAACTAGTTTATCGCGGGCTGCGTTTGGC (reverse). The forward primer includes the 3×-FLAG sequence (in bold) upstream of the natural RegA start codon. This amplified region was cloned into the construct described above using an *Nco*I site that overlaps with the start codon of the RegA gene and *Spe*I after the RegA stop codon. The overexpressed WT and RegA* constructs were designed with the same 3×-FLAG sequence and overlap as the WT construct, ordered from IDT and cloned into pSRKGm (Khan *et al.*, 2008). Both the 3×-FLAG WT RegA and the 3×-FLAG RegA* constructs were checked for functionality by complementation of the *regA* deletion strain DS05. Functional complementation was judged by restoration of photopigment production, which is repressed in DS05 due to loss of RegA (Fig. S1, available in the online Supplementary Material).

### ChIP-seq.

After addition of formaldehyde to a final concentration of 1 %, samples were incubated for 15 min at room temperature. Tris (pH 8.2) was then added to a final concentration of 500 mM and incubated for 5 min at room temperature. After centrifugation, cells were disrupted by two cycles of lysis via a French press followed by shearing of DNA using a small-tip sonicator at 15 W power. Chromatin precipitation was performed using ANTI-FLAG M2 Affinity Gel (Cat. No. A2220). After immunoprecipitation, crosslinking was reversed by incubation of samples at 65 °C for 1 h. RNA was removed by incubation with 1 µg RNAse A at 65 °C overnight. Samples were cleaned with a Qiagen MinElute Reaction Clean-up Kit with libraries made using the NEBNext Ultra DNA Library Prep Kit for Illumina and sequenced as above for RNA-seq. Data were trimmed using Trimmomatic with a sliding window of 5 : 25 and minimum read length of 40 bp. Trimmed reads were aligned to the *R. capsulatus* chromosome with Bowtie2 (Langmead & Salzberg, 2012) with peaks called using the MACS software package that also did not have corresponding peaks in a DNA only (ChIP with FLAG antibody in WT background) control (Zhang *et al.*, 2008).

### Comparison of *R. capsulatus* and *R. sphaeroides* regulons.

Orthologues of *R. capsulatus* in *R. sphaeroides* were found using the OMA orthology database, accessible at http://http://www.omabrowser.org (Altenhoff *et al.*, 2015).

## Results

### Overview of the number of genes regulated by RegA as assayed by RNA-Seq

Genes directly and indirectly regulated by RegA were identified by performing RNA-seq analyses of both aerobically and anaerobically (photosynthetically) grown WT cells as well as cells deleted for RegA (*ΔregA*). Differentially expressed genes (DEGs) were identified as those that exhibited a ≥2.0-fold change with adjusted *P*-value of ≤0.05 in pairwise comparison of the WT and *ΔregA* data sets from a minimum of three biological replicates on each assayed growth condition. *P*-values were calculated by the DESeq2 software package using the Wald test followed by the procedure of Benjamin and Hochberg to adjust for multiple testing (Love *et al.*, 2014). Validation of the RNA-seq datasets was performed by comparing RNA-seq determined fold changes of nine genes under varying conditions (20 samples in total) to fold changes found using qRT-PCR (Fig. S2). We undertook an analysis of WT–*ΔregA* changes in gene expression under both dark aerobic (respiratory) and illuminated anaerobic (photosynthetic) growth conditions in cells grown in rich growth medium (PY) and in cells grown in defined minimal malate medium (RCV) (Tables 1, S1 and S2).

**Fig. 1.**
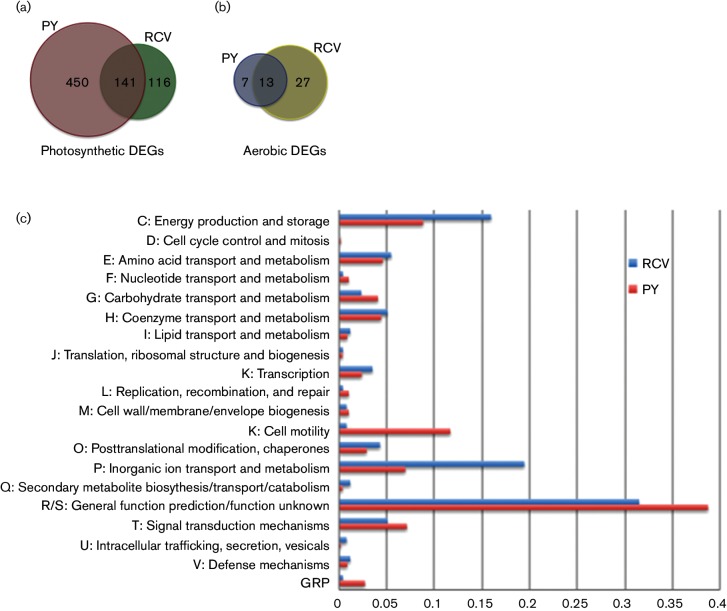
Venn diagrams showing the overlap between the identity of DEGs regulated ≥2-fold in photosynthetic and aerobically grown cells in PY and in RCV minimal malate medium. (a) Venn diagram representing the number of DEGs regulated by RegA when cells are grown in PY versus RCV medium under anaerobic photosynthetic conditions. (b) Same medium conditions with the exception that the cells were grown aerobically in the dark. (c) Bar chart of genes in the photosynthetic RegA regulon that are divided into COGs. Blue and red bars represent the percentage of genes in individual COG categories differentially expressed in cells grown under photosynthetic conditions in RCV medium or PY, respectively. COG GRP represents the cluster of genes that encode a glycyl radical enzyme-containing microcompartment.

The Venn diagrams in Fig. 1 provide an overview of the number of genes that undergo >2-fold change in expression upon the loss of RegA in rich and minimal growth medium under photosynthetic and aerobic conditions. Fig. 1(c) also provides a low-resolution analysis of the type of genes controlled by RegA by displaying the breakdown of DEGs in each cluster of orthologous groups (COG) (Fig. 1c is meant only as a means to display the proportion of genes in each COG relative to the total number of DEGs, not to invoke comparison between different COGs). Inspection of Fig. 1 shows that the number of genes regulated by RegA varies considerably depending on the medium that cells were grown in prior to RNA extraction. For example, 591 genes undergo >2-fold changes in expression upon loss of RegA when cells are grown under rich PY medium photosynthetic conditions (Fig. 1a). This contrasts with the 257 genes that undergo changes in expression upon loss of RegA in cells grown photosynthetically in RCV minimal malate medium (Fig. 1a). Interestingly, only 141 genes undergo changes in expression in both PY and minimal malate medium under photosynthetic conditions. This indicates that the majority of genes that are regulated by RegA under photosynthetic conditions are actually growth medium specific.

We also addressed the effect of oxygen on RegA-dependent gene expression. A previous study indicated that loss of RegA had an effect on aerobic expression of cytochrome *cbb*_3_ oxidase (Swem & Bauer, 2002), which was unexpected as the cognate sensor kinase RegB that phosphorylates RegA is thought to be active only under anaerobic conditions (Swem *et al.*, 2006; Wu & Bauer, 2008, 2010; Wu *et al.*, 2013). When assaying the effect that a loss of RegA had on aerobic gene expression, we observed that 40 genes exhibit >2-fold changes in expression in minimal malate medium while 20 genes showed >2-fold changes when grown aerobically in complex PY medium (Table 1 and Fig. 1b). As was the case with anaerobic expression, only a subset of these genes (13 genes) exhibit RegA-dependent aerobic changes in expression when grown in either PY or minimal malate medium.

**Table 1.** WT–*ΔregA* DEGs under aerobic conditions WT–*ΔregA* DEGs under aerobic conditions: DEGs under aerobic conditions in RCV and PY medium organized by COG and regulation strength. Genes highlighted in bold-face type indicate that they are regulated in both PY and RCV medium. −, repression; +, activation.

** Gene**	**RCV**		**PY**		**Regulation**
	**FC**	***P***	**FC**	***P***	
COG C: Energy production and storage					
**rcc03085 cydA; cytochrome d ubiquinol oxidase subunit I**	**−5.72**	**5.04E-153**	**−2.75**	**1.71E-41**	**+**
**rcc03084 cydB; cytochrome d ubiquinol oxidase subunit II**	**−4.34**	**9.20E-107**	**−3.90**	**6.01E-69**	**+**
rcc02015 aldH1; aldehyde dehydrogenase	−3.22	2.49E-64			+
**rcc01157 ccoN; cbb3-type cytochrome c oxidase subunit I**	**−2.80**	**3.72E-66**	**−2.66**	**3.02E-77**	**+**
**rcc01158 ccoO; cbb3-type cytochrome c oxidase subunit II**	**−2.99**	**3.37E-78**	**−2.74**	**6.74E-77**	**+**
**rcc01160 ccoP; cbb3-type cytochrome c oxidase subunit III**	**−2.98**	**2.20E-63**	**−2.76**	**7.56E-66**	**−**
**rcc01159 ccoQ; cbb3-type cytochrome c oxidase subunit IV**	**−2.91**	**2.32E-58**	**−2.68**	**6.74E-63**	**+**
**rcc01161 ccoG; cbb3-type cytochrome c oxidase accessory protein**	**−2.46**	**4.17E-58**	**−2.03**	**2.91E-30**	**+**
rcp00072 nosY; cooper ABC transporter permease	2.18	5.83E-20			−
rcp00070 nosX; NosX protein	2.24	1.83E-29			−
rcp00074 nosD; nitrous oxide maturation protein	2.31	6.16E-23			−
rcp00071 nosL; nitrous oxide reductase accessory protein	2.37	8.44E-26			**−**
rcp00073 nosF; copper ABC transporter ATP-binding protein	2.39	1.44E-22			**−**
rcp00075 nosZ; nitrous-oxide reductase	2.57	2.88E-46			−
					
COG E: Amino acid transport and metabolism					
rcc00516 tpl; tyrosine phenol-lyase	−2.38	1.54E-17			+
					
COG G: Carbohydrate transport and metabolism					
rcc03013 DeoC/LacD family aldolase	−2.28	2.65E-13			+
					
COG H: Coenzyme transport and metabolism					
rcc01596 cobA1; uroporphyrinogen-III C-methyltransferase			−2.49	6.15E-16	+
rcp00134 citG; triphosphoribosyl-dephospho-CoA synthase			−2.02	0.0001586203	+
					
COG K: Transcription					
rcc01722 BadM/Rrf2 family transcriptional regulator	−3.13	1.05E-33			+
rcp00076 nosR; nitrous-oxide reductase expression regulator	2.25	2.54E-21			−
					
COG Q: Secondary metabolites biosythesis, transport and catabolism					
rcc02016 fumarylacetoacetate hydrolase	−3.19	2.20E-63			+
					
COG R/S: General function prediction only/function unknown					
**rcc02390 alkane 1-monooxygenase**	**−7.46**	**9.62E-66**	**−3.51**	**2.39E-20**	**+**
**rcc03083 membrane bound YbgT-like protein**	**−3.44**	**1.34E-23**	**−2.58**	**1.49E-09**	**+**
rcc00435 hypothetical protein	−3.06	2.35E-17			+
rcc02463 hemolysin-type calcium-binding repeat family protein	−2.54	3.32E-20			+
rcc03377 hypothetical protein	−2.43	1.49E-19	−2.42	3.00E-20	+
rcc03467 hypothetical protein	−2.35	5.54E-07	−2.22	0.0002062298	+
rcc00542 hypothetical protein	−2.05	2.06E-11			+
rcc00747 hypothetical protein			−2.12	0.0004716659	+
rcc00979 hypothetical protein			−2.23	1.64E-05	+
rcc02367 calcium-binding protein			−2.54	3.74E-09	+
rcp00031 phage integrase			−2.07	0.0001252058	+
rcp00068 M4 family peptidase	2.02	9.06E-11			−
rcc01027 hypothetical protein	2.05	4.03E-07			−
rcc00048 metal dependent phosphohydrolase	2.09	1.71E-21			−
rcc02119 type 12 family methyltransferase	2.31	2.39E-05			−
rcc03429 hypothetical protein	2.59	1.40E-54			−
rcp00077 hypothetical protein	2.65	8.00E-22			−
rcc01849 hypothetical protein	2.95	2.53E-76			−
rcp00069 hypothetical protein	2.73	6.88E-37	2.30	1.74E-08	−
rcc02118 cat; chloramphenicol acetyltransferase	4.70	4.27E-78	2.84	7.33E-18	**−**
					
COG T: Signal transduction mechanisms					
**rcc00045 regA1; photosynthetic apparatus regulatory protein**	**−12.73**	**3.64E-47**	**−68.75**	**0**	+
+rcc02857 diguanylate cyclase/phosphodiesterase	−2.07	4.31E-31			+
rcc02856 PAS/PAC sensor domain-containing protein	−2.02	3.15E-15			+
rcc02849 dorS; DMSO/TMAO-sensor hybrid histidine kinase	2.03	1.41E-10			−
rcc01156 UspA domain-containing protein	2.10	1.45E-16			−

### Members of the RegA regulon that are altered in response to different nutrient conditions

**Fig. 2.**
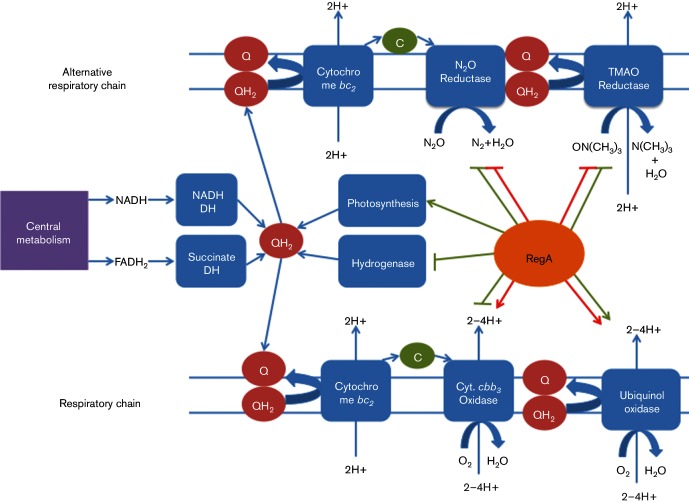
RegA control of *R. capsulatus* respiratory pathways. Electron flow during aerobic and anaerobic respiratory growth. RegA positively regulates terminal oxidases and negatively regulates alternative terminal electron acceptors in the presence of oxygen (red arrows). In this way electron flow towards terminal oxidases is favoured. In the absence of oxygen (green arrows), RegA positively regulates photosynthesis genes and the ubiquinol oxidase, while negatively regulating the hydrogenase complex, cytochrome *cbb_3_* oxidase and the two alternative terminal electron acceptors.

Inspection of the group of 141 genes that exhibit altered photosynthesis expression in both rich and minimal medium conditions (Fig. 1a and highlighted in bold type in Tables S1 and S2) shows that many previously identified RegA-regulated genes are encompassed by this group. These include such notable genes as the photosystem structural genes coded by the *puf* and *puc* operons, *hup* genes coding for structural and maturation components of hydrogenase, *cco* genes coding for structural and assembly components of the respiratory cytochrome *cbb_3_* oxidase, *fdh* genes coding for subunits of NAD-dependent formate dehydrogenase, *nifA* coding for a nitrogen-specific regulatory protein and *hem* genes coding for enzymes involved in heme biosynthesis. Each of these has been shown to be regulated by RegA with many involving direct regulation as based on DNAseI footprint studies (Fig. 2) (Du *et al.*, 1998; Bird *et al.*, 1999; Elsen *et al.*, 2000; Vichivanives *et al.*, 2000; Hemschemeier *et al.*, 2000; Swem *et al.*, 2001; Willett *et al.*, 2007). New notable RegA-regulated genes that exhibit changes in expression in both rich and minimal medium include *nosYFDZ* (rcc00072–rcc00075) which code for subunits of the nitrous oxide reductase, several subunits of an iron siderophore/cobalamin ABC transporter (rcc03359, rcc03360 and rcc10028) and several genes involved in iron uptake (*rcc_001028–rcc_01030*, and *EfeUBO*, *feoA1A2B1*, *hmuVR* and *rcc_03358–rcc_03361*). There are also several regulatory genes exhibiting altered expression in both rich PY and minimal malate growth media such as a *σ*^54^ family sigma factor (rcc02453), a signal transduction histidine kinase (rcc02294) and several diguanylate cyclase/phosphodiesterases (rcc01020, rcc00643, rcc02857) (Tables S1 and S2).

Analysis of differentially expressed genes present only in the photosynthetic minimal medium data set (116 genes; Table S2) also revealed several newly identified RegA-regulated genes including those coding for lactate, succinate and glycolate dehydrogenases that could affect the cell’s reducing potential (Table S2, COG C). There are also numerous genes involved in polyamine and amino acid transport (Table S2, COG E) and genes involved in heme and iron transport (Table S2, COG P). Finally, several genes involved in signal transduction (Table S2, COG T) and numerous transcription factors (Table S2, COG K) are regulated by RegA in minimal medium.

Detailed analysis of iron transport expression revealed 40 genes related to iron uptake that are anaerobically repressed by RegA in minimal medium (Table S2, COG P). These genes include *hmuVUTSR* (hemin transport), *fhuC1B1B2D1* (ferrichrome transport), *fepB1D1G1C2G2D2B2* (ferric enterobactin transport), *efeUO* (ferrous iron transport) and iron siderophore/cobalamin transport genes (*rcc01028*, *rcc01046* and *rcc01047*). Three genes related to iron transport are also activated during photosynthetic growth in minimal malate medium, *rcc_03358–rcc-03360*, which code for an iron siderophore/cobalamin ABC transporter complex. *R. capsulatus* also contains an ‘iron island’ from *rcc00091–rcc00112* which contains a ferrous iron transporter *feoA_1_A_2_BC*, a heme transporter *hmuRSTUV*, an ABC transporter (*rcc00099–rcc00103*), an iron siderophore uptake system *fhuE-fes-fhuDB_1_B_2_C_1_* and an AraC-family transcriptional regulator *rcc00112* (Zappa & Bauer, 2013). Our analysis indicates that RegA represses all of these genes with the exception of the *feo* operon. Rodionov *et al.* (2006) reported that four AraC-like transcriptional regulators in *R. capsulatus* (*rcc_00012*, *rcc_01048*, *rcc_01431* and *rcc_01432*) are believed to control iron uptake. Three of these (*rcc_00012*, *rcc_01048* and *rcc_01432*) are repressed by RegA. Given that the minimal medium contains 14 µM Fe^2+^ in its composition and PY medium contains ~3 µM iron, the observed repression of numerous iron transport genes by RegA indicates that iron is provided in excess in this medium and that RegA appears to have an important role in the control of iron homeostasis.

Analysis of the large group of 450 genes that exhibit differential expression only in rich PY medium (Fig. 1 and Table S1) shows that a large number of motility genes (69) are regulated by RegA (Table S1, COG N). This includes 29 genes related to flagella biosynthesis (*flgABCDEFGHIJKL*, *fliEFGHIL1L2NPQR*, *flhAB*, *flaAF*, *flbT*, *motAB* and *rcc_03522*), nine genes related to gas vesicle formation (*gvpAGKJON*, *rcc_01056*, *rcc_01058* and *rcc_01060*), 15 methyl-accepting chemotaxis proteins (*mcpA1A2A3BCHIX*, *rcc_01075*, *rcc_01185*, *rcc_01355*, *rcc_01667*, *rcc_02151*, *rcc_02887* and *rcc_03014*) and 14 chemotaxis genes (*cheY1A1W1WR2B1* and *cheB2DY2R3W2A2Y3X* operons). With the exception of one methyl-accepting chemotaxis protein (*rcc_*02151), differential expression of motility genes is not observed when cells are grown anaerobically in minimal malate medium. To test whether *ΔregA* cells are indeed defective in motility, we performed soft PY agar stab motility assays with WT, *ΔregA* and *ΔflaA* cells (Fig. S3). As shown in this figure, *ΔregA* cells resemble the non-motile *ΔflaA* cells in PY medium, while WT cells exhibit significant motility.

**Fig. 3.**
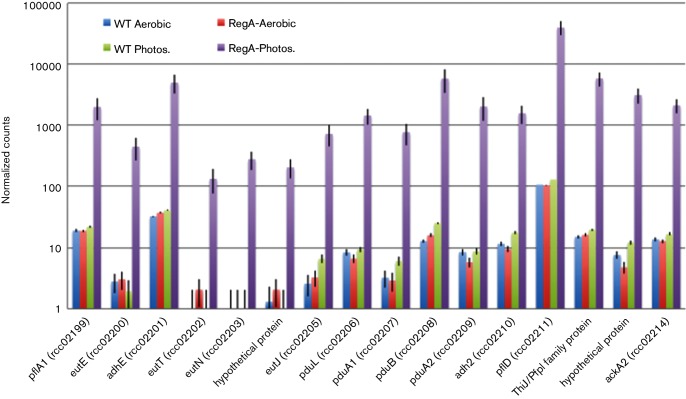
GRP microcompartment. Normalized gene counts are shown from RNA-seq experiments in PY medium with photosynthetically and aerobically grown WT and *ΔregA* cells. Blue and green bars show gene expression for WT cells during aerobic and photosynthetic growth, respectively. Red and purple bars show gene expression for *ΔregA* cells during aerobic and photosynthetic growth, respectively.

Also preferentially expressed in PY medium under anaerobic conditions is a cluster of 15 genes (*rcc_02199rcc_02213*) (COG GRP in Table S1) that exhibit the highest level of differential expression changes in the cell ranging from 76- to 250-fold (these genes are anaerobically repressed by RegA) (Fig. 3). Analysis of this gene cluster indicates that they code for a bacterial microcompartment containing a putative 1,2-propanediol (1,2-PD) breakdown pathway (Jorda *et al.*, 2013; Axen *et al.*, 2014; Zarzycki *et al.*, 2015). This pathway is related to a previously characterized *pdu* 1,2-PD breakdown microcompartment that utilizes a B_12_-dependent diol dehydratase enzyme. However, a notable change exists with the *R. capsulatus* gene cluster in that it codes for a B_12_-independent glycyl radical enzyme (Jorda *et al.*, 2013; Axen *et al.*, 2014; Zarzycki *et al.*, 2015). Fig. 3 shows the normalized gene counts and annotated functions for the 15 genes under photosynthetic conditions in both media with *pdu* referring to genes that share homology with genes involved in 1,2—PD breakdown and *eut* for those involved in ethanolamine breakdown, respectively. This log scale graph shows that members of this gene cluster increase expression several orders of magnitude higher in a RegA deletion strain grown anaerobically in PY medium.

### RegA regulation of genes under aerobic conditions

A total of 40 genes were differentially expressed under aerobic conditions in malate minimal medium (Table 1). Of these, 22 had lower expression in *ΔregA* cells than in WT cells while 18 had higher expression. Of genes with known function, 14 belong to the COG C, which includes genes for energy conversion and storage, including *cydAB* (ubiquinol oxidase) and *ccoNOPQG* (cytochrome *cbb*_3_ oxidase) which have previously been reported to be aerobically regulated by RegA (Swem & Bauer, 2002). Additional genes that are aerobically repressed include nitrous oxide reductase (*nosXLYFDZ*), *nosR*, which regulates transcription of the *nos* operon, and *dorS*, which regulates the transcription of the DMSO/TMAO reductase complex. Both *nosR* and *dorS* are repressed by RegA and control the expression of proteins that function as alternative terminal electron acceptors (Fig. 2). Finally, five genes are aerobically regulated that belong to COG K or T involved in transcription and signalling, respectively.

When grown under aerobic conditions in PY medium, 18 genes were activated and two repressed by RegA (Table 1). These include seven genes in COG C that code for components of cytochrome *cbb*_3_ oxidase (*ccoNOPQG*) and ubiquinol oxidase (*cydAB*). Both of these sets of respiratory genes are activated by RegA when grown in both PY and minimal malate medium. The remaining genes were assigned to COG R/S (nine genes) with the exception of one gene in COG H, *cobA1*. The two repressed genes, *rcp00069* and *cat*, are either uncharacterized or code for a chloramphenicol acetyltransferase, respectively.

When comparing the aerobic data sets with the anaerobic data sets we observed that of the 40 DEGs under aerobic minimal medium conditions, all but three are also regulated by RegA under photosynthetic minimal medium conditions. The genes *dorS*, *rcc01849* (both repressed) and *rcc3083* (activated) are the only ones that are differentially expressed only under aerobic conditions. It is also interesting that the aerobically activated genes are also activated under photosynthetic conditions, with the exception of the *cco* operon which is repressed under photosynthetic conditions. All DEGs that are repressed aerobically are also repressed photosynthetically.

### Chromatin immunoprecipitation analysis of RegA binding sites

We addressed which genes are directly controlled by RegA by performing ChIP-seq analysis. For this analysis, we utilized two different 3×FLAG-RegA constructs. One construct expressed 3×FLAG-RegA from its native promoter. The second construct expressed a mutant variant of 3×FLAG-RegA called 3×FLAG-RegA* + HvrA that contains a point mutation in RegA that renders RegA constitutively active even in the absence of phosphorylation (Du *et al.*, 1998; Bird *et al.*, 1999) as well as HvrA, which is an HN-S family protein that is transcriptionally coupled with RegA (Buggy *et al.*, 1994).

**Fig. 4.**
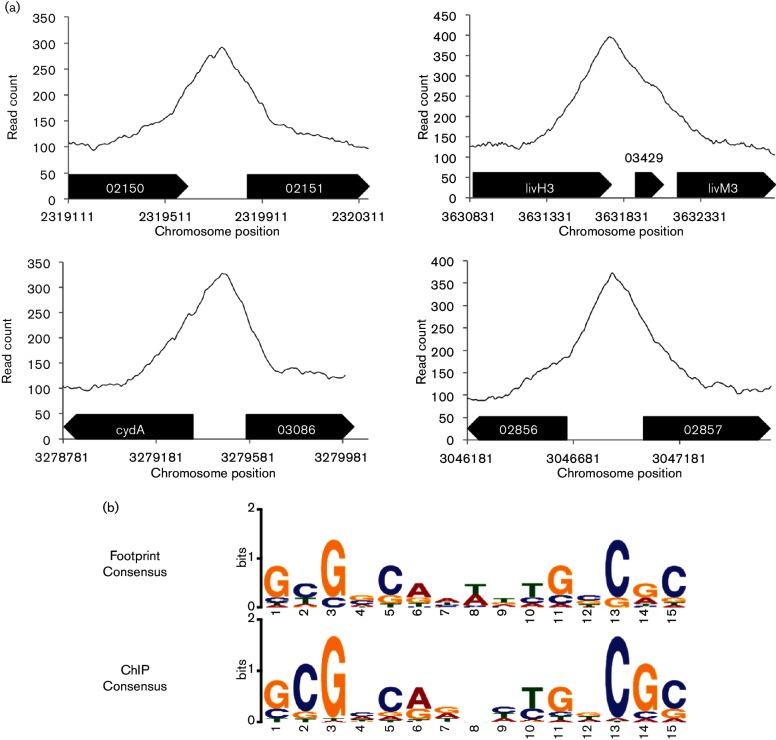
ChIP-seq overview. (a) Four representative peaks along with their chromosomal context. The genes represented are *rcc_02150* (*ancA* aconitate hydratase), *rcc_02151* (methyl-accepting chemotaxis sensory transducer), *livH3* (branched chain amino acid transporter permease), *rcc_03429* (putative membrane protein), *livM3* (branched chain amino acid transporter permease), *cydA* (cytochrome d ubiquinol oxdase subunit I), *rcc_03086* (hypothetical protein), *rcc_02856* (PAS/PAC sensor domain protein)and *rcc_02857* (diguanylate cyclase/phosphodiesterase with PAS/PAC sensor). (b) Comparison of RegA binding sequence motifs from 20 previously identified DNase I footprints and this study.

Peaks generated from deep sequencing of the 3×FLAG-RegA immunoprecipated DNA relative to a sheared genomic DNA input control were called as RegA binding sites using MACS model-based analysis with a p-value cutoff of 1.00e^−5^ with representative examples shown in Fig. 4. There were 61 unique peaks called across all four datasets (anaerobic PY and malate minimal medium with WT RegA and anaerobic PY and malate minimal medium with RegA* plus HvrA). A majority of these peaks (35) were present in both the WT RegA data set and the RegA* plus HvrA data set while 13 called peaks were present only in the WT RegA data set and 13 only in the RegA* plus HvrA data sets. Of the 61 called RegA binding sites, 33 had corresponding changes in gene expression in the RNA-seq data sets (Table 2). Among this group, 18 were present in both PY and minimal malate while seven peaks were present in PY alone and eight only in RCV minimal medium.

**Table 2.** ChIP peaks with corresponding changes in gene expression Genes highlighted in bold-face type indicate that they are regulated in both PY and RCV medium. NCP, no called peak; N/A, not applicable. −, repression; +, activation.

**Peak**	**Start**	**End**	**Sequence**	**ChIP fold enrich.**		**Gene(s)**	**Annotation**	**RNA-seq FC**		**Regulation**
				**RCV**	**PY**			**PY**	**RCV**	
1	191712	193420	GCACCATTCCAACGG	NCP	2.48	*rcc_00165*	NAD-dependent epimerase/dehydratase	−2.01	N/A	+
2	452768	455370	CGGTCAGATTGACGC	3.03	2.97	*rcc_00423*	*hypothetical protein*	**6.28**	**2.46**	**−**
3	465189	466644	GCGACAGGACGCCGC	5.57	NCP	*rcc_00435*	*hypothetical protein*	N/A	**−2.28**	+
4	580707	581931	TCGCCATGACGGCGA	NCP	2.47	*rcc_00542*	*hypothetical protein*	−3.55	−5.63	+
						*rcc_00543*	*hypothetical protein*	N/A	−2.48	+
5	756419	765975	TCTTGAAATGGTCGA	3.14	2.39	*crtF*	*carotenoid biosynthesis*	**−2.92**	N/A	+
						*pufQ*	*cytochrome*	**−2.06**	**−2.23**	+
						*pufB*	*light harvesting complex*	**−2.05**	**−2.41**	+
						*pufA*	*light harvesting complex*	**−2.01**	**−2.32**	+
						*pufL*	*reaction center*	**−2.29**	**−2.33**	+
						*pufM*	*reaction center*	**−2.28**	**−2.33**	+
6	803297	809177	GGGATAGTTTGTCGC	3.45	2.15	*sdhB*	*succinate dehydrogenase*	2.25	N/A	−
7	833738	834931	CCGCGGGGCTGTCCC	3.32	3.46	*hoxH*	*NAD-reducing hydrogenase subunit beta*	2.96	N/A	−
						*hoxW*	*hydrogenase maturation factor*	N/A	**2.05**	**−**
8	988257	989655	GGAAGATTACGACGC	5.18	2.89	*rcc_00901*	*hypothetical*	2.75	N/A	−
9	1225054	1230563	GCGACCGTTCGTCGC	5.13	4.05	*rcc_01156*	*universal stress protein*	**14.15**	**3.70**	**−**
						*ccoN*	*cytochrome cbb3 oxidase*	**12.48**	**8.39**	**−**
						*ccoO*	*cytochrome cbb3 oxidase*	**12.75**	**9.66**	**−**
						*ccoP*	*cytochrome cbb3 oxidase*	**11.37**	**9.00**	**−**
						*ccoQ*	*cytochrome cbb3 oxidase*	**13.08**	**10.07**	**−**
10	1254741	1255894	GCGATGGCCTTGCGG	NCP	2.12	*rcc_001184*	*CsbD family protein*	2.26	**N/A**	**−**
11	1413837	1415609	GCGAGGGACCGGCGC	2.23	NCP	*rcc_01319*	*hypothetical protein*	2.74	**N/A**	**−**
12	1564402	1565565	CCGAATTCTTGACGA	NCP	2.59	*hemA*	*5-aminolevulinate synthase*	**−2.59**	**−2.04**	+
13	1619610	1622691	GCTGCAAAACGACGG	4.12	2.87	*fusA2*	*translation elongation factor G*	**7.00**	**2.69**	**−**
14	1861705	1868009	GCGACATGGCCACGC	3.96	2.55	*rcc_01729*	*pyridine nucleotide-disulfide oxidoreductase*	5.32	N/A	**−**
						*nifJ*	*pyruvate-flavodoxin oxidoreductase*	**8.76**	**3.67**	**−**
						*pyrD1*	*dihydroorotate oxidase*	**2.90**	**2.07**	**−**
15	2048221	2049528	GCGCCCGACTGGCGC	NCP	2.98	*rcc_01897*	*hypothetical protein*	7.30	**N/A**	**−**
16	2161963	2163124	CCGTCATTTTGGCGG	NCP	2.18	*rcc_02005*	*hypothetical protein*	**N/A**	−3.37	+
17	2319111	2320348	CTGCCAAAATGTCGC	4.97	NCP	*rcc_02151*	*methyl-accepting chemotaxis sensory transducer*	**−13.21**	**−4.25**	+
18	2494714	2495999	GCGCGTAAACGACCC	4.75	2.49	*rcc_02321*	*hypothetical*	3.22	N/A	−
19	2661146	2662930	TGGCTGATCTGACGA	4.85	3.02	*rcc_02479*	*cytochrome c*	2.90	N/A	**−**
20	2705099	2706586	GCGGAGGACTGTCGC	2.37	NCP	*rcc_02520*	*integrin alpha/hemolysin-type calcium-binding* *repeat*	2.86	N/A	**−**
21	2719251	2722948	AGGTCGAATTGTCGC	4.67	2.61	*pucB*	*light harvesting complex*	**−5.32**	**−5.34**	+
						*pucA*	*light harvesting complex*	**−5.63**	**−3.59**	+
						*pucC2*	*light harvesting complex*	**−3.94**	**−3.78**	+
						*pudCE*	*light harvesting complex*	**−2.87**	**−3.30**	+
22	2778625	2780581	GCTTCGATCCGGCGC	4.96	4.13	*fadJ*	*fatty acid oxidation complex*	2.98	N/A	**−**
						*dksA2*	*dnaK suppressor protein*	2.86	N/A	**−**
23	2785733	2787067	GCGAAAACCCGGCGC	3.46	NCP	*rcc_02597*	*hypothetical protein*	−3.40	N/A	**+**
24	2873668	2876166	GCACCGAACCGGCGC	4.79	2.35	*rcc_02683*	*cheR methyltransferase*	2.29	N/A	**−**
						*rcc_02684*	*polyphosphate 2 domain-containing protein*	4.06	N/A	**−**
25	2952271	2953727	TCGAGGAATTGCCGC	5.23	3.83	*rcc_02764*	*hypothetical*	11.59	N/A	**−**
26	3039970	3041450	GCTTGGGGTTGGCGC	2.53	2.66	*dorS*	*DMSO/TMAO sensor histidne kinase*	2.92	N/A	**−**
27	3046184	3047609	CGGACAAAATGTCGC	6.22	NCP	*rcc_02856*	*PAS/PAC sensor domain-containing protein*	**−34.31**	**−2.46**	+
						*rcc_02857*	*diguanylate cyclase/phosphodiesterase*	**−12.81**	**−2.04**	+
28	3059338	3063326	TCGTCAAGCTGACGC	3.59	2.21	*pflB*	*formate C-acetyltransferase*	4.27	N/A	−
						*pflA2*	*pyruvate formate lyase-activating*	5.18	N/A	−
29	3278779	3280086	GTGACAATTTGTCCC	5.74	NCP	*cydA*	*ubiquinol oxidase*	N/A	−2.30	+
						*cydB*	*ubiquinol oxidase*	N/A	−2.12	+
30	3551924	3553160	GTGCAAGTCCGGCGC	NCP	2.2	*rcc_03356*	*hypothetical protein*	−2.82	N/A	+
31	3630831	3632812	GCGTCAAAGTGTCCA	6.22	2.23	*rcc_03429*	*hypothetical*	**5.20**	**5.87**	**−**
						*livM3*	*branched chain amino acid transporter permease*	**4.59**	**N/A**	**−**
32	p. 54259	p. 55549	GCGTCAATATGGCGC	5.23	NCP	*rcp_00068*	*M4 family peptidase*	**3.43**	**2.92**	**−**
						*rcp_00069*	*hypothetical*	**9.28**	**7.35**	**−**
33	p. 114642	p. 116478	GCGTCAAAGCGCCGC	5.67	3.21	*citG*	*triphosphoribosyl-dephospho-CoA synthase*	**−4.46**	**−2.00**	+

### Comparison with *R. sphaeroides* PrrA regulon

A similar microarray and ChIP-seq study was recently performed with the RegA homologue PrrA from *R. sphaeroides* by Imam *et al.* (2014). In their study, they assayed the effect of a RegA deletion on gene expression in cells grown under anaerobic conditions in minimal medium with succinate as a carbon source and DMSO as a terminal electron acceptor [this growth condition was used as *ΔprrA* cells cannot grow photosynthetically (Imam *et al.*, 2014)]. The authors observed that expression of 255 genes was affected by deletion of *R. sphaeroides* PrrA, which is similar to the number of genes affected upon deletion of the *R. capsulatus* RegA (257 genes) when growing anaerobically (photosynthetically) in minimal malate medium. However, inspection of individual genes affected by RegA and PrrA show that the vast majority differ. Specifically, among the 257 genes that are regulated by *R. capsulatus* RegA (based on a fold change of >2.0), only 23 are also affected upon deletion of *R. sphaeroides* PrrA (Table S4). Inspection of the 23 genes that are regulated in common by RegA and PrrA shows that they include the photosystem structural genes coded by the *puf* and *puc* operons, heme biosynthesis genes coded by *hemA* and *hemE*, and respiratory genes coded by *ccoN* and *ccoQ*. The number of overlapping genes increases slightly to 39 if the stringency is decreased to a fold change >1.5, with the inclusion of several additional photosynthesis-related genes such as *bchZID* and *crtEA*, *hemC*, *cytB*, and carbon fixation genes *cbbPM* and *tkt1*.

A comparison of RegA binding sites determined by ChIP-seq shows that only nine of the 61 binding sites found in *R. capsulatus* are also present upstream of similar genes in the *R. sphaeroides* PrrA ChIP-seq data set. As with the limited overlap in DEGs, the binding sites shared by both species are upstream of photosystem structural genes and a heme biosynthesis gene (Table S4).

## Discussion

### Variability of RegA-regulated genes under different nutrient conditions

A major conclusion is that the number of genes affected upon deletion of RegA varies considerably in response to nutrient growth conditions. Although PY is considered a ‘rich’ medium, research has shown that these types of media have very few sugars and instead offer amino acids as the main carbon source (Sezonov *et al.*, 2007). In contrast, RCV minimal medium used in this study has malate as a sole carbon source. One can imagine that growth in PY medium requires cells to change their metabolism as amino acids are undergoing transport and breakdown as a food source, versus in RCV minimal medium where amino acids need to be synthesized. This could be an explanation for the larger number of DEGs found during growth in PY medium versus minimal malate medium and also the large increase in DEGs in COG-E (Amino Acid Transport and Metabolism) seen in PY. The minimal medium that was employed also contained excess iron. Consequently, WT cells appear to use RegA to limit iron transport in minimal medium.

Previous studies have shown that RegA functions as an accessory activator of energy-generating and energy-utilizing processes that have additional dedicated transcription factors. For example, RegA is needed for optimal expression of genes involved in carbon and nitrogen fixation, the utilization of hydrogen as an energy source and the use of DMSO as a terminal electron acceptor (Richardson *et al.*, 1991, 1994; Elsen *et al.*, 2000; Kappler *et al.*, 2002; Vichivanives *et al.*, 2000; McCrindle *et al.*, 2005). However, each of these systems also have dedicated transcription factors that respond to carbon, nitrogen, hydrogen and DMSO/TMAO availability, respectively. It is therefore easy to envisage why there are significant differences in the number of genes affected by RegA between rich and minimal medium as growth in these very different nutrient conditions probably utilizes vastly different metabolic and catabolic pathways each of which has different transcription factors that supersede or recruit the involvement of RegA. As evidence of this, Table S3 shows that there are far more genes in COGs K and T regulated above fourfold in rich medium (25 genes) than in minimal medium (six genes). One example of this type of regulation involves regulation of the Calvin–Benson–Bassham (CBB) CO_2_ fixation pathway by the dedicated carbon sensing regulator CbbR and RegA (Dubbs *et al.*, 2000; Vichivanives *et al.*, 2000; Gibson *et al.*, 2002). Activation of *cbb* gene expression involves both transcription factors, with RegA stimulating the binding of CbbR to the *cbb* promoter region (Dangel & Tabita, 2009; Dangel *et al.*, 2014). Interestingly, it has been shown that the DNA binding motif of RegA directly interacts with the DNA binding motif of CbbR (Dangel *et al.*, 2014) and that RegA can interact with CbbR in the absence of a DNA template (Dangel & Tabita, 2009). Additional detailed analyses of RegA interaction with other transcription factors, such as those involved in nitrogen fixation and the utilization of hydrogen, have not been undertaken but it is likely that similar complex interactions of RegA occur with additional transcription factors leading to growth medium variability in the number of genes that exhibit alerted expression upon deletion of RegA.

### Regulation under aerobic conditions

While it was previously reported that RegA aerobically regulates the expression of the ubiquinol and cytochrome *cbb_3_* terminal oxidases (Swem & Bauer, 2002), it was not known that RegA also aerobically controls the expression of nitrous oxide reductase and DMSO/TMAO reductase. Nitrous oxide and DMSO/TMAO reductases are both utilized during anaerobic respiration so it is not surprising that RegA represses expression when oxygen is present for aerobic respiration (Fig. 2). It was previously known that RegA represses the *dor* operon under photoheterotrophic conditions with malate as a carbon source (Kappler *et al.*, 2002), but regulation under aerobic conditions has not been reported.

It is unclear how this control is achieved because RegB is not thought to be phosphorylated by RegB under aerobic conditions. The observation that RegA can function as an aerobic activator and also as an anaerobic repressor of *cbb_3_* oxidase has led to the model that unphosphorylated RegA and phosphorylated RegA may both be capable of altering gene expression by binding to different binding sites (Swem *et al.*, 2001). This phenomenon has also been observed in the similar ArcB/ArcA two-component systemin *Escherichia coli*, where ArcA activates expression of the succinate dehydrogenase complex during aerobic growth and represses it during anaerobic growth (Shen & Gunsalus, 1997). It is also worth noting that many genes regulated aerobically are also regulated under photosynthetic conditions, indicating that RegA may be a constitutive regulator of these genes regardless of phosphorylation.

### Comparison of PrrA and RegA

Comparison of DEGs regulated by *R. capsulatus* RegA with *R. sphaeroides* PrrA shows very few genes in common beyond photosynthesis and respiration. Analysis of orthologues between these species shows that of 3493 protein-coding genes in the *R. capsulatus* genome only 2092 have orthologues in the *R. sphaeroides* genome. The number of genes regulated by RegA under photosynthetic RCV conditions was 257, only 94 of which have orthologues in *R. sphaeroides*; among these, 23 are regulated by both RegA and PrrA with the majority directly related to photosynthesis and respiration (Table S4). When viewing the type of genes that are regulated by RegA and PrrA, a few patterns appear. RegA activates and represses many more genes in COG P category ‘Inorganic Transport and Metabolism’ than does PrrA. This category includes ~40 genes related to iron transport, an operon related to vitamin B_12_ transport (*rcc_03358–03360*) and an operon (*rcc_01647*, *rcc_01648*, *rcc_01650*, *rcc_01651*) coding for NitT/TauT transport family members that transport nitrites/nitrates and taurines. There is also a large difference in COG category C ‘Energy Production and Storage’ where *R. sphaeroides* PrrA represses many genes involved in the TCA cycle while *R. capsulatus* RegA does not. PrrA also activates more genes in COG H ‘Coenzyme Transport and Metabolism’ than does RegA and also activates a significant number of motility genes in minimal medium (COG category N), which is contrasted by *R. capsulatus* RegA, which activates motility genes when cells are grown in PY medium.

What is the mechanism of divergence in the type of genes regulated by *R. capsulatus* RegA and *R. sphaeroides* PrrA? RegA and PrrA exhibit a high degree of sequence similarity (83 %) with 100 % conservation in the helix–turn–helix DNA binding motif (Elsen *et al.*, 2004). Furthermore, *R. capsulatus* RegA complements an *R. sphaeroides* PrrA deletion in regard to photosystem pigmentation and the regulation of carbon fixation (*cbb*) gene expression (Vichivanives *et al.*, 2000). This suggests that RegA and PrrA are functionally equivalent and that the mechanism of divergence between these species probably lies in the location of RegA and PrrA recognition sequences. This conclusion is supported by similar analysis of the FnrL in *R. capsulatus* and *R. sphaeroides* using both RNA-seq and ChIP-seq (Imam *et al.*, 2014; Kumka & Bauer, 2015). Similar to RegA, there is a high degree of sequence similarity between FnrL orthologues (78 %) and yet out of 807 genes that are regulated by *R. capsulatus* FnrL (and 917 *R. sphaeroides* FnrL) only 171 are commonly regulated across these species. The divergence is even more stark when comparing the location of ChIP-seq identified FnrL binding sites where there are only nine FnrL binding sites (out of 81 called peaks) present upstream of orthologous genes between these species (Kumka & Bauer, 2015). Clearly, analysis of orthologous transcription factors in these different species shows considerable divergence in the nature of genes that are being regulated. It may be of interest to undertake similar analysis with additional strains from these species that have been isolated from different habitats to determine if the observed divergence in RegA and FnrL target locations remains. It would also be of interest to determine if different strains in the same species retain the same target gene, which would address whether target divergence is a species-driven event.

## Supplementary Data

765 Supplementary File 1
