# Supplementary material for: The RegA regulon exhibits variability in response to altered growth conditions and differs markedly between Rhodobacter species
Source: Microb Genom. 2016 Oct 21;2(10):e000081. doi: 10.1099/mgen.0.000081 (PMC5359404; doi:10.1099/mgen.0.000081)
Supplement: Supplementary File 1 [file mgen-02-81-s001.pdf]

Supplementary Data

Figure S1. Spectral scans of light harvesting photopigments from photosynthetically grown *Rba. capsulatus* cells harvested at mid-log phase. The 800 and 865 nm peaks represent light harvesting I and II absorption from wild type SB1003 cells (blue). These peaks are repressed in the *regA* deletion strain DS05 (red) but fully complimented by the M2-FLAG-RegA (green) and M2-FLAG-RegA* expressing plasmids.

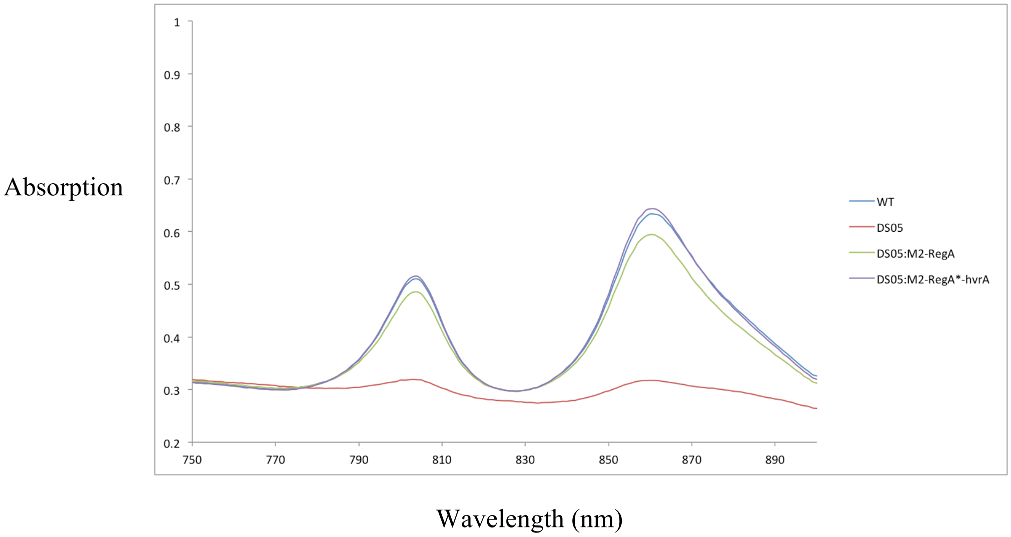

Figure S2. RT-PCR validation of RNAseq experiments. a) Table showing the fold changes found for nine genes under varying conditions using RNA-seq and RT-PCR. b) Correlation between fold changes found for genes tested in A. c) Bar graph showing the comparison between fold changes found for genes listed in A. Blue bars represent RNA-seq fold changes and red bars represent RT-PCR fold changes.

a

Gene	Condition	RNA-seq FC	RNA-seq pval	RT-PCR FC	RT-PCR pval	Agree?
**ccoN**	RCV Aer	-2.96	0.000	-2.70	0.048	Yes
	RCV PS	9.24	0.000	14.02	0.065	Yes
	PY Aer	-2.65	0.000	-2.45	0	Yes
	PY PS	12.41	0.000	17.95	0.017	Yes
**nosL**	RCV Aer	2.38	0.000	1.16	0.501	Yes
	RCV PS	13.74	0.000	14.43	0.032	Yes
	PY Aer	1.44	0.014	2.83	0.032	Yes
	PY PS	4.22	0.001	4.41	0	Yes
**pucB**	RCV PS	-4.36	0.000	-2.49	0	Yes
	PY PS	-5.48	0.000	-10.10	0.082	Yes
**pufM**	RCV PS	-2.33	0.000	-1.90	0.032	Yes
	PY PS	-2.28	0.000	-3.29	0.082	Yes
**petB**	RCV PS	-1.47	0.000	-1.30	0.136	Yes
	PY PS	-1.22	0.126	-2.34	0.081	Yes
**hemA**	RCV PS	-2.04	0.000	2.05	0.085	No
	PY PS	-2.58	0.000	-1.73	0.374	Yes
**puhA**	RCV PS	-1.50	0.000	1.67	0.054	No
	PY PS	-1.22	0.134	-1.72	0.141	Yes
**rcc_02197**	PY PS	7.73	0.000	4.19	0.012	Yes
**rcc_02198**	PY PS	5.66	0.000	3.42	0	Yes

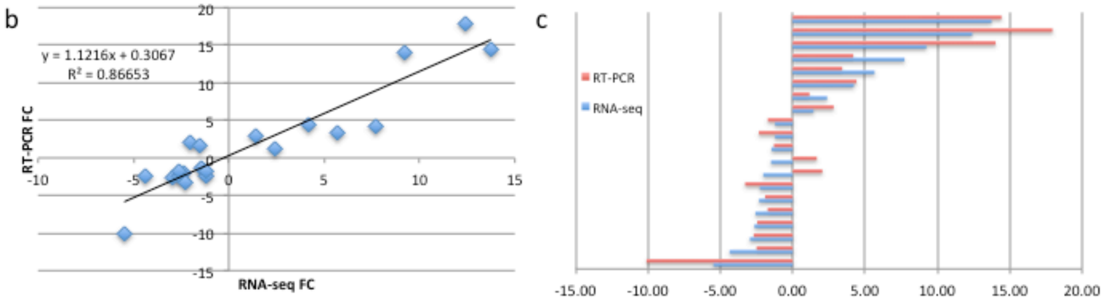

Fig. S3. Motility: A soft-agar stab assay in PY media shows motility of wild-type cells, non-motile Δ*flaA* cells, and Δ*regA* cells.

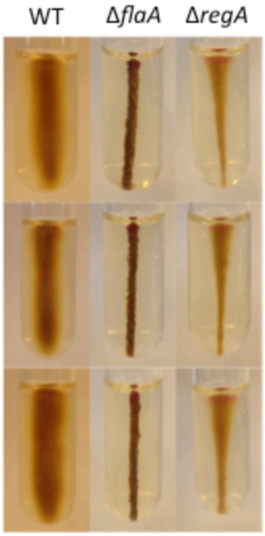

Table S1: WT-Δ*regA* DEGs under photosynthetic conditions in PY medium

Gene		COG	FC	p-val
COG C: Energy production and storage				
rcp:RCAP_rcc00436	cytB; cytochrome B561; K12262 cytochrome b561	C	-2.39	1.76E-11
**rcp:RCAP_rcc00690**	**pufQ; cytochrome, subunit PufQ**	**C**	**-2.06**	**2.10E-07**
**rcp:RCAP_rcc00691**	**pufB; light-harvesting protein B-870 subunit beta**	**C**	**-2.05**	**5.50E-14**
**rcp:RCAP_rcc00692**	**pufA; light-harvesting protein B-870 subunit alpha**	**C**	**-2.01**	**2.57E-15**
**rcp:RCAP_rcc00693**	**pufL; photosynthetic reaction center subunit L**	**C**	**-2.29**	**7.31E-14**
**rcp:RCAP_rcc00694**	**pufM; photosynthetic reaction center subunit M**	**C**	**-2.28**	**1.62E-14**
**rcp:RCAP_rcc00695**	**pufX; intrinsic membrane protein PufX**	**C**	**-2.27**	**4.57E-14**
rcp:RCAP_rcc00869	adhC; bifunctional alcohol dehydrogenase	C	-2.01	2.15E-05
**rcp:RCAP_rcc01646**	**cytochrome c domain-containing protein**	**C**	**-14.02**	**5.46E-07**
**rcp:RCAP_rcc02015**	**aldH1; aldehyde dehydrogenase**	**C**	**-10.96**	**1.28E-139**
**rcp:RCAP_rcc02530**	**pucB; light-harvesting protein B-800/850 subunit beta**	**C**	**-5.32**	**3.35E-79**
**rcp:RCAP_rcc02531**	**pucA; light-harvesting protein B-800/850 subunit alpha**	**C**	**-5.63**	**3.96E-79**
**rcp:RCAP_rcc02532**	**pucC2; protein PucC; K08226 MFS transporter**	**C**	**-3.94**	**3.35E-16**
**rcp:RCAP_rcc02533**	**pucDE; light-harvesting protein B-800/850 subunit gamma**	**C**	**-2.87**	**1.20E-20**
**rcp:RCAP_rcc01656**	**cycA2; cytochrome c2**	**C**	**-2.61**	**0.000180098**
**rcp:RCAP_rcc02702**	**cytochrome c/b561 family protein**	**C**	**-3.82**	**5.94E-18**
**rcp:RCAP_rcc03016**	**draT; NAD(+)--dinitrogen-reductase ADP-D-ribosyltransferase**	**C**	**-2.66**	**0.000106567**
**rcp:RCAP_rcc00022**	**aldo/keto reductase family oxidoreductase**	**C**	**2.08**	**4.73E-06**
rcp:RCAP_rcc00736	sdhB; succinate dehydrogenase, iron-sulfur subunit	C	2.25	2.19E-17
rcp:RCAP_rcc00761	hoxH; NAD-reducing hydrogenase HoxS subunit beta	C	2.96	1.05E-12
**rcp:RCAP_rcc00767**	**hupA; hydrogenase small subunit**	**C**	**7.76**	**1.74E-33**
**rcp:RCAP_rcc00768**	**hupB; hydrogenase large subunit**	**C**	**9.67**	**6.86E-56**
**rcp:RCAP_rcc00769**	**hupC; hydrogenase, cytochrome b subunit**	**C**	**11.05**	**6.49E-67**
**rcp:RCAP_rcc01157**	**ccoN; cbb3-type cytochrome c oxidase subunit I**	**C**	**12.48**	**6.23E-73**
**rcp:RCAP_rcc01158**	**ccoO; cbb3-type cytochrome c oxidase subunit II**	**C**	**12.75**	**3.96E-59**
**rcp:RCAP_rcc01159**	**ccoQ; cbb3-type cytochrome c oxidase subunit IV**	**C**	**13.08**	**8.00E-62**
**rcp:RCAP_rcc01160**	**ccoP; cbb3-type cytochrome c oxidase subunit III**	**C**	**11.37**	**5.79E-46**
**rcp:RCAP_rcc01161**	**ccoG; cbb3-type cytochrome c oxidase accessory protein CcoG**	**C**	**4.55**	**5.25E-35**
**rcp:RCAP_rcc01162**	**ccoH; cbb3-type cytochrome c oxidase biogenesis protein CcoH**	**C**	**2.77**	**6.41E-22**
**rcp:RCAP_rcc01163**	**ccoI; cbb3-type cytochrome c oxidase biogenesis protein CcoI**	**C**	**2.70**	**1.72E-20**
rcp:RCAP_rcc01533	nuoJ; NADH-quinone oxidoreductase subunit J	C	2.06	4.45E-10
**rcp:RCAP_rcc01728**	**nifJ; pyruvate-flavodoxin oxidoreductase**	**C**	**8.76**	**1.40E-24**
rcp:RCAP_rcc01729	pyridine nucleotide-disulfide oxidoreductase	C	5.32	1.63E-16
rcp:RCAP_rcc02248	aldo/keto reductase family oxidoreductase	C	5.23	4.67E-19
rcp:RCAP_rcc02653	glpK1; glycerol kinase	C	3.57	2.07E-25
rcp:RCAP_rcc02845	torA; trimethylamine-N-oxide reductase	C	33.85	5.68E-103
rcp:RCAP_rcc02846	torD; chaperone protein TorD	C	34.07	9.59E-23
rcp:RCAP_rcc02847	torC; trimethylamine-N-oxide reductase c-type cytochrome TorC	C	31.65	7.88E-84
rcp:RCAP_rcc02866	pflB; formate C-acetyltransferase	C	4.27	1.61E-11
rcp:RCAP_rcc02869	glcF; glycolate dehydrogenase, iron-sulfur subunit	C	2.96	6.67E-18
rcp:RCAP_rcc02870	glcE; glycolate dehydrogenase, subunit GlcE	C	3.79	2.93E-17
rcp:RCAP_rcc02871	glcD; glycolate dehydrogenase, subunit GlcD	C	2.63	1.63E-11
rcp:RCAP_rcc03034	fdhD; formate dehydrogenase accessory protein FdhD	C	3.81	1.16E-05
**rcp:RCAP_rcc03035**	**fdhA; NAD-dependent formate dehydrogenase subunit alpha**	**C**	**3.28**	**2.07E-15**
**rcp:RCAP_rcc03036**	**fdhB; NAD-dependent formate dehydrogenase subunit beta**	**C**	**2.47**	**2.32E-08**
**rcp:RCAP_rcc03037**	**fdhC; NAD-dependent formate dehydrogenase subunit gamma**	**C**	**2.10**	**1.97E-06**
rcp:RCAP_rcc03048	lldD; L-lactate dehydrogenase	C	2.30	2.86E-13
rcp:RCAP_rcc03350	ferredoxin domain-containing protein oxidireductase	C	3.72	2.62E-14
**rcp:RCAP_rcp00072**	**nosY; cooper ABC transporter permease NosY**	**C**	**3.32**	**5.99E-06**
**rcp:RCAP_rcp00073**	**nosF; copper ABC transporter ATP-binding protein NosF**	**C**	**4.23**	**0.000537817**
**rcp:RCAP_rcp00074**	**nosD; nitrous oxide maturation protein NosD**	**C**	**4.01**	**0.001024362**
**rcp:RCAP_rcp00075**	**nosZ; nitrous-oxide reductase**	**C**	**3.81**	**0.009649827**

COG D: Cell cycle control and mitosis

rcp:RCAP_rcc03418	smc; chromosome partition protein Smc	D	-2.61	3.44E-28

COG E: Amino acid transport and metabolism

rcp:RCAP_rcc00336	bztB; glutamate/aspartate ABC transporter permease BztB	E	-3.06	1.02E-15
rcp:RCAP_rcc00337	bztC; glutamate/aspartate ABC transporter permease BztC	E	-2.68	1.90E-13
rcp:RCAP_rcc01072	hutH; histidine ammonia-lyase	E	-8.98	4.39E-50
rcp:RCAP_rcc01243	potA1; polyamine ABC transporter ATP binding protein PotA	E	-2.11	0.000881392
rcp:RCAP_rcc01244	potD1; polyamine ABC transporter periplasmic polyamine-binding protein F	E	-2.08	0.003665553
rcp:RCAP_rcc01245	potB1; polyamine ABC transporter permease PotB	E	-2.09	0.007563141
rcp:RCAP_rcc01543	ilvI; acetolactate synthase large subunit	E	-2.19	8.12E-13
rcp:RCAP_rcc02267	potB3; polyamine ABC transporter permease PotB	E	-2.26	6.11E-06
rcp:RCAP_rcc02268	potD3; polyamine ABC transporter periplasmic polyamine-binding protein F	E	-2.30	0.001163986
rcp:RCAP_rcc03353	metH1; methionine synthase subunit A	E	-2.03	0.004496779
rcp:RCAP_rcc03428	livH3; branched-chain amino acid ABC transporter permease LivH	E	-2.56	0.002082038
rcp:RCAP_rcp00004	polar amino acid ABC transporter ATP-binding protein	E	-2.00	0.001357397
**rcp:RCAP_rcc00516**	**tpl; tyrosine phenol-lyase**	**E**	**-8.92**	**2.52E-49**
rcp:RCAP_rcc00409	AzlC family protein	E	2.19	1.78E-12
**rcp:RCAP_rcc00484**	**ald; alanine dehydrogenase**	**E**	**3.70**	**3.41E-26**
rcp:RCAP_rcc01096	abgB; aminobenzoyl-glutamate utilization protein B	E	3.21	2.84E-33
rcp:RCAP_rcc01218	ureA; urease subunit gamma	E	2.08	0.003387108
rcp:RCAP_rcc01219	ureB; urease subunit beta	E	2.63	0.00015283
rcp:RCAP_rcc02274	pip; proline iminopeptidase	E	6.80	1.72E-19
rcp:RCAP_rcc02275	oppA2; oligopeptide ABC transporter periplasmic oligopeptide-binding prot	E	7.15	5.80E-53
rcp:RCAP_rcc02276	oppB2; oligopeptide ABC transporter permease OppB	E	3.04	1.23E-13
rcp:RCAP_rcc02277	oppC2; oligopeptide ABC transporter permease OppC	E	3.00	9.42E-12
rcp:RCAP_rcc02278	oppD2; oligopeptide ABC transporter ATP-binding protein OppD	E	2.73	4.23E-10
rcp:RCAP_rcc02313	soxG; sarcosine oxidase subunit gamma .	E	2.27	9.44E-08
**rcp:RCAP_rcc02451**	**potA4; polyamine ABC transporter ATP-binding protein PotA**	**E**	**2.11**	**0.007283065**
**rcp:RCAP_rcc02452**	**potD4; polyamine ABC transporter periplasmic polyamine-binding proteir**	**E**	**3.55**	**7.67E-13**
rcp:RCAP_rcc03430	livM3; branched-chain amino acid ABC transporter permease LivM	E	4.59	2.45E-15

COG F: Nucleotide transport and metabolism

**rcp:RCAP_rcc03092**	**apt; adenine phosphoribosyltransferase**	**F**	**-2.89**	**1.02E-10**
rcp:RCAP_rcc03342	nrdD; anaerobic ribonucleoside-triphosphate reductase	F	-2.48	2.02E-09
rcp:RCAP_rcc00790	xdhC; xanthine dehydrogenase accessory protein XdhC	F	2.64	5.99E-09
rcp:RCAP_rcc00791	xdhB; xanthine dehydrogenase molybdopterin binding subunit	F	2.30	2.30E-15
**rcp:RCAP_rcc01727**	**pyrD1; dihydroorotate oxidase**	**F**	**2.90**	**4.07E-08**
rcp:RCAP_rcc02617	ushA; 5'-nucleotidase	F	2.58	1.06E-18

COG G: Carbohydrate transport and metabolism

rcp:RCAP_rcc00165	NAD-dependent epimerase/dehydratase	G	-2.01	6.03E-07
**rcp:RCAP_rcc01657**	**exaA2; quinoprotein ethanol dehydrogenase**	**G**	**-4.03**	**1.97E-07**
rcp:RCAP_rcc01831	gap1; glyceraldehyde-3-phosphate dehydrogenase	G	-2.20	6.13E-06
rcp:RCAP_rcc01832	tkt1; transketolase	G	-2.03	0.001955781
rcp:RCAP_rcc02411	edd; phosphogluconate dehydratase	G	-2.13	0.000169608
**rcp:RCAP_rcc03013**	**DeoC/LacD family aldolase**	**G**	**-4.48**	**4.79E-32**
rcp:RCAP_rcc00253	major facilitator superfamily protein	G	2.35	4.33E-11
rcp:RCAP_rcc00576	cbbO; rubisco activation protein CbbO	G	2.88	6.01E-12
rcp:RCAP_rcc00577	cbbQ; CbbQ protein	G	2.86	3.24E-06
rcp:RCAP_rcc00578	cbbS; ribulose bisphosphate carboxylase small subunit	G	2.99	1.83E-06
rcp:RCAP_rcc00579	cbbL; ribulose bisphosphate carboxylase large subunit	G	2.70	3.10E-05

rcp:RCAP_rcc01771	aglE; alpha-glucoside ABC transporter substrate-binding protein	G	2.10	1.65E-10
rcp:RCAP_rcc01772	aglF; alpha-glucoside ABC transporter permease	G	2.24	1.18E-11
rcp:RCAP_rcc01773	aglG; alpha-glucoside ABC transporter permease	G	2.28	5.12E-14
rcp:RCAP_rcc01774	aglA; alpha-glucosidase	G	2.58	8.26E-20
rcp:RCAP_rcc01775	aglK; alpha-glucoside ABC transporter ATP-binding protein	G	2.94	1.54E-22
rcp:RCAP_rcc02373	monosacharide ABC transporter periplasmic monosacharide-binding protei	G	2.37	5.30E-08
rcp:RCAP_rcc02652	RbsD/FucU transport protein family	G	2.52	1.19E-19
rcp:RCAP_rcc02654	tktB; transketolase, C-terminal subunit	G	4.08	7.30E-38
rcp:RCAP_rcc02655	tktA; transketolase, N-terminal subunit	G	3.83	4.13E-36
rcp:RCAP_rcc02656	hypothetical protein	G	3.74	3.41E-43
**rcp:RCAP_rcc02657**	**monosacharide ABC transporter periplasmic monosacharide-binding prot**	**G**	**6.13**	**3.51E-89**
**rcp:RCAP_rcc02660**	**monosacharide ABC transporter permease**	**G**	**4.77**	**9.61E-60**
rcp:RCAP_rcc03049	major facilitator superfamily protein	G	2.23	1.88E-11

COG GRP: Glycyl radical enzyme microcompartment

rcp:RCAP_rcc02199	pflA1; [pyruvate formate-lyase]-activating enzyme	GRP	88.70	5.14E-100
rcp:RCAP_rcc02200	hypothetical protein	GRP	76.33	3.10E-55
rcp:RCAP_rcc02201	adhE; aldehyde-alcohol dehydrogenase	GRP	103.54	1.06E-148
rcp:RCAP_rcc02202	hypothetical protein	GRP	84.38	8.95E-28
rcp:RCAP_rcc02203	eutN; ethanolamine utilization protein EutN	GRP	110.29	1.05E-42
rcp:RCAP_rcc02204	hypothetical protein	GRP	110.00	1.10E-32
rcp:RCAP_rcc02205	eutJ; ethanolamine utilization protein EutJ	GRP	129.92	2.72E-71
rcp:RCAP_rcc02206	pduL; propanediol utilization protein PduL	GRP	141.22	7.07E-109
rcp:RCAP_rcc02207	pduA1; propanediol utilization protein PduA	GRP	110.84	1.62E-72
**rcp:RCAP_rcc02208**	**pduB; propanediol utilization protein PduB**	**GRP**	**196.48**	**5.05E-141**
rcp:RCAP_rcc02209	pduA2; propanediol utilization protein PduA	GRP	112.32	2.12E-37
rcp:RCAP_rcc02210	adh2; aldehyde-alcohol dehydrogenase 2	GRP	97.00	4.55E-100
rcp:RCAP_rcc02211	pflD; formate C-acetyltransferase	GRP	251.31	0
rcp:RCAP_rcc02212	ThiJ/PfpI family protein; K05520 protease I	GRP	248.49	2.41E-191
rcp:RCAP_rcc02213	hypothetical protein	GRP	207.04	2.42E-149
rcp:RCAP_rcc02214	ackA2; acetate kinase	GRP	96.18	3.60E-133

COG H: Coenzyme transport and metabolism

rcp:RCAP_rcc00671	bchG; bacteriochlorophyll synthase	H	-2.09	1.09E-08
rcp:RCAP_rcc00679	crtI; phytoene dehydrogenase	H	-2.44	2.77E-22
rcp:RCAP_rcc00680	crtB; phytoene synthase	H	-2.00	3.10E-15
rcp:RCAP_rcc00684	crtE; farnesyltranstransferase	H	-2.59	5.24E-12
rcp:RCAP_rcc00685	crtF; hydroxyneurosporene methyltransferase	H	-2.92	1.94E-10
rcp:RCAP_rcc01034	CbiM family cobalamin biosynthesis protein	H	-2.49	7.30E-09
rcp:RCAP_rcc01120	metK; methionine adenosyltransferase	H	-2.05	1.96E-09
**rcp:RCAP_rcc01172**	**hemE; uroporphyrinogen decarboxylase**	**H**	**-2.79**	**7.80E-17**
**rcp:RCAP_rcc01447**	**hemA; 5-aminolevulinate synthase**	**H**	**-2.59**	**6.60E-07**
**rcp:RCAP_rcc03091**	**mtnP; S-methyl-5-thioadenosine phosphorylase**	**H**	**-3.24**	**1.68E-14**
rcp:RCAP_rcc03354	cobQ2; cobyric acid synthase CobQ	H	-2.81	7.82E-14
rcp:RCAP_rcc03355	cobQ3; cobyric acid synthase CobQ	H	-2.51	1.83E-10
**rcp:RCAP_rcc03362**	**cbiZ; adenosylcobinamide amidohydrolase**	**H**	**-3.56**	**0.00014612**
**rcp:RCAP_rcc01235**	**pyrimidine 5'-nucleotidase**	**H**	**-4.27**	**4.84E-15**
**rcp:RCAP_rcc00028**	**idi1; isopentenyl-diphosphate delta-isomerase**	**H**	**-8.33**	**3.84E-24**
**rcp:RCAP_rcp00134**	**citG; triphosphoribosyl-dephospho-CoA synthase**	**H**	**-4.46**	**0.014103395**
**rcp:RCAP_rcc02430**	**nahG; salicylate hydroxylase**	**H,C**	**-2.24**	**1.00E-13**
**rcp:RCAP_rcc01644**	**FMN-binding domain-containing protein**	**H,C,K**	**-2.88**	**2.07E-06**
rcp:RCAP_rcc00622	moeA; molybdopterin biosynthesis protein MoeA	H	2.16	4.02E-09
**rcp:RCAP_rcc00712**	**moaA1; molybdenum cofactor biosynthesis protein A**	**H**	**2.06**	**1.72E-06**
**rcp:RCAP_rcc02638**	**calcium-binding EF-hand domain-containing protein**	**H**	**2.25**	**1.27E-05**
rcp:RCAP_rcc02840	moaC2; molybdenum cofactor biosynthesis protein C	H	8.33	1.06E-22

rcp:RCAP_rcc02841	moeB2; molybdenum cofactor biosynthesis protein B	H	16.27	2.20E-53
rcp:RCAP_rcc02842	moaD1; molybdenum cofactor biosynthesis protein D	H	18.00	2.99E-22
rcp:RCAP_rcc02843	moaA2; molybdenum cofactor biosynthesis protein A	H	14.47	2.45E-53
rcp:RCAP_rcc03261	TOBE domain-containing protein	H	3.52	6.10E-31

COG I: Lipid transport and metabolism

rcp:RCAP_rcc01063	pcl; 4-coumarate--CoA ligase	I	-4.62	1.78E-09
rcp:RCAP_rcc02126	acsA1; acetate--CoA ligase	I	-3.27	0.01127976
**rcp:RCAP_rcc02620**	**acsA2; acetate--CoA ligase**	**I**	**-3.65**	**2.12E-36**
rcp:RCAP_rcc01515	hmgL; hydroxymethylglutaryl-CoA lyase	I	2.08	0.00138684
rcp:RCAP_rcc02589	fadJ; fatty acid oxidation complex subunit alpha	I	2.98	1.58E-13

COG J: Translation, ribosomal structure and biogenesis

rcp:RCAP_rcc00536	GNAT family acetyltransferase	J	-2.15	9.61E-12
rcp:RCAP_rcc00118	sigma 54 modulation protein/ribosomal protein S30EA	J	2.38	2.79E-20

COG K: Transcription

rcp:RCAP_rcc00112	AraC family transcriptional regulator	K	-2.52	0.022497672
rcp:RCAP_rcc00550	LysR family transcriptional regulator	K	-3.59	2.04E-13
**rcp:RCAP_rcc00568**	**rpoN; RNA polymerase sigma-54 factor**	**K**	**-2.04**	**0.001250995**
rcp:RCAP_rcc01100	XRE family transcriptional regulator	K	-2.18	8.67E-07
**rcp:RCAP_rcc01722**	**BadM/Rrf2 family transcriptional regulator**	**K**	**-3.66**	**3.47E-10**
rcp:RCAP_rcc02460	cspA2; cold shock protein CspA	K	-2.34	4.25E-10
rcp:RCAP_rcc02675	MarR family transcriptional regulator	K	-7.78	4.94E-88
rcp:RCAP_rcc03298	LysR family transcriptional regulator	K	-2.08	2.09E-05
rcp:RCAP_rcc00574	Crp/Fnr family transcriptional regulator	K	2.00	0.006681275
rcp:RCAP_rcc01130	LuxR family transcriptional regulator	K	3.27	1.19E-22
rcp:RCAP_rcc02291	ECF family RNA polymerase sigma factor	K	2.69	4.90E-09
**rcp:RCAP_rcc02453**	**Fis family sigma54 specific transcriptional regulator**	**K**	**5.13**	**6.84E-11**
**rcp:RCAP_rcp00076**	**nosR; nitrous-oxide reductase expression regulator NosR**	**K**	**5.03**	**3.10E-20**
rcp:RCAP_rcp00084	ArsR family transcriptional regulator	K	2.34	0.001202595

COG L: Replication, recombination, and repair

rcp:RCAP_rcc00222	radC; DNA repair protein RadC; K03630 DNA repair protein RadC	L	-2.30	1.45E-11
rcp:RCAP_rcc00463	hypothetical protein; K07460 putative endonuclease	L	-2.64	8.96E-13
rcp:RCAP_rcc01298	SNF2 family helicase (EC:3.6.1.-)	L	-2.34	3.42E-06
rcp:RCAP_rcc02742	cytosine-specific DNA-methyltransferase (EC:2.1.1.37); K00558 DNA (cytosi	L	-2.35	0.000118583
rcp:RCAP_rcc03098	drpA; DNA protecting protein DprA; K04096 DNA processing protein	L	-2.87	1.63E-10
rcp:RCAP_rcc02864	PAS domain/exonuclease domain-containing protein; K02342 DNA polymer	L	8.69	1.38E-08

COG M: Cell wall/membrane/envelope biogenesis

rcp:RCAP_rcc00055	transglycosylase, Slt family	M	-21.57	7.75E-71
rcp:RCAP_rcc00614	RND efflux system outer membrane lipoprotein	M	-2.71	2.07E-08
rcp:RCAP_rcc01697	NlpC/P60 family phage cell wall peptidase	M	-4.70	0.00368448
**rcp:RCAP_rcc00887**	**hypothetical protein**	**M**	**6.03**	**4.02E-26**
rcp:RCAP_rcc01949	undecaprenyl-phosphate galactosephosphotransferase (EC:2.7.8.6)	M	3.38	2.29E-24
rcp:RCAP_rcc03420	LrgB family protein	M	3.01	2.64E-24

COG N: Cell motility

rcp:RCAP_rcc00006	motB; chemotaxis protein MotB	N	-2.96	1.96E-29
rcp:RCAP_rcc00007	flgE; flagellar hook protein FlgE	N	-11.48	2.56E-130

rcp:RCAP_rcc00008	flgK; flagellar hook-associated protein FlgK	N	-7.26	1.82E-76
rcp:RCAP_rcc00009	flgL; flagellar hook-associated protein FlgL	N	-7.73	7.72E-89
rcp:RCAP_rcc00010	flgI; flagellar P-ring protein FlgI	N	-6.80	4.30E-106
rcp:RCAP_rcc00056	flhA; flagellar biosynthesis protein FlhA	N	-29.15	5.62E-163
rcp:RCAP_rcc00057	fliR; flagellar biosynthetic protein FliR	N	-20.17	2.82E-106
rcp:RCAP_rcc00058	flhB; flagellar biosynthetic protein FlhB	N	-15.81	1.73E-97
rcp:RCAP_rcc00481	mcpI; methyl-accepting chemotaxis protein McpI	N	-2.97	7.42E-24
rcp:RCAP_rcc00644	mcpX; methyl-accepting chemotaxis protein McpX	N	-4.87	1.14E-23
rcp:RCAP_rcc00759	mcpB; methyl-accepting chemotaxis protein McpB	N	-20.15	4.70E-165
rcp:RCAP_rcc00760	mcpA1; methyl-accepting chemotaxis protein McpA	N	-14.20	1.40E-102
rcp:RCAP_rcc01051	gvpN; gas vesicle protein GvpN	N	-7.76	6.55E-31
rcp:RCAP_rcc01053	gvpO; gas vesicle protein GvpO	N	-8.18	3.20E-11
rcp:RCAP_rcc01054	gvpJ; gas vesicle protein GvpJ	N	-6.97	5.55E-13
rcp:RCAP_rcc01056	gas vesicle synthesis protein GvpL/GvpF	N	-6.45	3.82E-19
rcp:RCAP_rcc01057	gvpG; gas vesicle protein GvpG	N	-5.25	4.65E-12
rcp:RCAP_rcc01058	gas vesicle synthesis protein GvpL/GvpF	N	-3.91	2.30E-05
rcp:RCAP_rcc01060	gas vesicle protein GvpA	N	-3.75	0.010561144
rcp:RCAP_rcc01062	gvpK; gas vesicle protein GvpK	N	-2.42	0.006527279
rcp:RCAP_rcc01073	gvpA; gas vesicle protein GvpA	N	-10.08	1.76E-40
rcp:RCAP_rcc01075	methyl-accepting chemotaxis protein	N	-6.09	2.41E-26
rcp:RCAP_rcc01185	methyl-accepting chemotaxis sensory transducer	N	-2.94	1.17E-05
rcp:RCAP_rcc01352	cheY1; chemotaxis protein CheY	N	-6.92	5.04E-34
rcp:RCAP_rcc01353	cheA1; chemotaxis protein CheA	N	-7.28	1.55E-40
rcp:RCAP_rcc01354	cheW1; chemotaxis protein CheW	N	-8.19	9.25E-39
rcp:RCAP_rcc01355	methyl-accepting chemotaxis sensory transducer	N	-7.86	4.60E-73
rcp:RCAP_rcc01356	chemotaxis protein CheW	N	-6.86	4.25E-39
rcp:RCAP_rcc01357	cheR2; chemotaxis protein methyltransferase CheR	N	-7.20	2.22E-51
rcp:RCAP_rcc01358	cheB1; chemotaxis response regulator protein-glutamate methylesterase C	N	-5.28	3.79E-26
rcp:RCAP_rcc01621	mcpC; methyl-accepting chemotaxis protein McpC	N	-2.54	1.81E-18
rcp:RCAP_rcc01667	methyl-accepting chemotaxis sensory transducer	N	-3.50	3.58E-32
rcp:RCAP_rcc01726	mcpH; methyl-accepting chemotaxis protein McpH	N	-3.93	1.35E-11
rcp:RCAP_rcc01758	mcpA2; methyl-accepting chemotaxis protein McpA	N	-9.19	3.20E-102
rcp:RCAP_rcc01759	cheB2; chemotaxis response regulator protein-glutamate methylesterase C	N	-13.13	3.16E-86
rcp:RCAP_rcc01760	cheD; chemoreceptor glutamine deamidase CheD	N	-9.32	9.87E-47
rcp:RCAP_rcc01762	cheY2; chemotaxis protein CheY	N	-13.06	3.55E-100
rcp:RCAP_rcc01763	cheR3; chemotaxis protein methyltransferase CheR	N	-15.47	2.10E-132
rcp:RCAP_rcc01764	cheW2; chemotaxis protein CheW	N	-14.44	1.69E-132
rcp:RCAP_rcc01765	cheA2; chemotaxis protein CheA	N	-15.50	2.92E-161
rcp:RCAP_rcc01766	cheY3; chemotaxis protein CheY	N	-19.95	3.90E-176
rcp:RCAP_rcc01767	cheX; chemotaxis protein CheX	N	-21.75	1.34E-139
**rcp:RCAP_rcc02151**	**methyl-accepting chemotaxis sensory transducer**	**N**	**-13.21**	**9.27E-71**
rcp:RCAP_rcc02158	fliG; flagellar motor switch protein FliG	N	-6.16	6.93E-58
rcp:RCAP_rcc02611	mcpA3; methyl-accepting chemotaxis protein McpA	N	-10.12	2.23E-100
rcp:RCAP_rcc02887	methyl-accepting chemotaxis sensory transducer	N	-8.33	2.18E-73
rcp:RCAP_rcc03014	methyl-accepting chemotaxis sensory transducer	N	-20.44	7.73E-189
rcp:RCAP_rcc03479	fliP; flagellar biosynthetic protein FliP	N	-3.07	1.34E-36
rcp:RCAP_rcc03480	fliN; flagellar motor switch protein FliN	N	-7.45	1.67E-42
rcp:RCAP_rcc03481	fliH; flagellar biosynthesis/type III secretory pathway protein FliH	N	-11.28	3.03E-75
rcp:RCAP_rcc03482	fliF; flagellar M-ring protein FliF	N	-10.05	7.28E-166
rcp:RCAP_rcc03483	fliL1; flagellar basal body-associated protein FliL	N	-14.34	4.19E-117
rcp:RCAP_rcc03486	motA; chemotaxis protein MotA	N	-10.85	2.93E-146
rcp:RCAP_rcc03512	fliL2; flagellar basal body-associated protein FliL	N	-6.23	1.56E-66
rcp:RCAP_rcc03513	flgH; flagellar L-ring protein FlgH	N	-10.94	7.15E-118
rcp:RCAP_rcc03514	flgA; flagella basal body P-ring formation protein FlgA	N	-11.50	2.89E-89
rcp:RCAP_rcc03515	flgG; flagellar basal-body rod protein FlgG	N	-12.51	2.33E-117
rcp:RCAP_rcc03516	flgF; flagellar basal-body rod protein FlgF	N	-12.10	1.85E-128
rcp:RCAP_rcc03517	fliQ; flagellar biosynthetic protein FliQ	N	-10.84	6.02E-103

rcp:RCAP_rcc03518	fliE; flagellar hook-basal body complex protein FliE	N	-9.40	3.57E-102
rcp:RCAP_rcc03519	flgC; flagellar basal-body rod protein FlgC	N	-10.09	1.20E-107
rcp:RCAP_rcc03520	flgB; flagellar basal-body rod protein; FlgB	N	-10.79	3.00E-118
rcp:RCAP_rcc03521	fliI; flagellar protein export ATPase FliI	N	-6.73	2.49E-58
rcp:RCAP_rcc03522	flagellar protein	N	-16.38	3.50E-114
rcp:RCAP_rcc03523	flbT; flagellin synthesis repressor protein FlbT	N	-22.91	3.49E-176
rcp:RCAP_rcc03524	flagellar FlaF family protein	N	-18.48	6.98E-134
rcp:RCAP_rcc03525	flaA; flagellin protein	N	-10.51	7.91E-126
rcp:RCAP_rcc03527	flgJ; flagellar protein FlgJ	N	-5.09	1.26E-39
rcp:RCAP_rcc03529	flgD; flagellar hook capping protein	N	-5.47	2.70E-59

COG O: Posttranslational modification, protein turnover, chaperones

rcp:RCAP_rcc02062	Hsp70 family heat shock protein	O	-2.34	2.24E-08
rcp:RCAP_rcc02069	hypothetical protein	O	-15.08	3.84E-104
**rcp:RCAP_rcc00640**	**S1/S6 family peptidase**	**O**	**2.55**	**2.67E-05**
**rcp:RCAP_rcc00762**	**hoxW; hydrogenase maturation factor HowW**	**O**	**3.37**	**3.93E-15**
**rcp:RCAP_rcc00770**	**hupD; hydrogenase maturation protease HupD**	**O**	**3.04**	**7.82E-18**
**rcp:RCAP_rcc00771**	**hupF; hydrogenase maturation chaperone HupF**	**O**	**4.61**	**1.61E-09**
**rcp:RCAP_rcc00772**	**hupG; hydrogenase expression/formation protein HupG**	**O**	**3.90**	**1.56E-23**
**rcp:RCAP_rcc00773**	**hupH; hydrogenase expression/formation protein HupH**	**O**	**4.77**	**2.85E-50**
**rcp:RCAP_rcc00774**	**hupJ; rubredoxin HupJ**	**O**	**3.81**	**5.36E-38**
**rcp:RCAP_rcc00775**	**hupK; hydrogenase expression/formation protein HupK**	**O**	**4.77**	**1.29E-19**
rcp:RCAP_rcc00776	hypA; hydrogenase nickel incorporation protein HypA	O	3.31	1.78E-20
**rcp:RCAP_rcc00777**	**hypB; hydrogenase nickel incorporation protein HypB**	**O**	**3.39**	**1.08E-33**
rcp:RCAP_rcc00779	hypC; hydrogenase assembly chaperone HypC/HupF	O	3.27	1.07E-14
**rcp:RCAP_rcc00780**	**hypD; hydrogenase expression/formation protein HypD**	**O**	**3.13**	**6.37E-23**
rcp:RCAP_rcc00781	hypE; hydrogenase expression/formation protein HypE	O	2.17	2.97E-05
rcp:RCAP_rcc02865	pflA2; [pyruvate formate-lyase]-activating enzyme	O	5.18	1.06E-13
rcp:RCAP_rcp00082	thioredoxin family protein	O	2.52	0.00012054

COG P: Inorganic ion transport and metabolism

rcp:RCAP_rcc00090	feoA1; ferrous iron transport protein A	P	-2.35	0.00055511
rcp:RCAP_rcc00091	feoA2; ferrous iron transport protein A	P	-2.45	0.000102234
rcp:RCAP_rcc00092	feoB1; ferrous iron transport protein B	P	-2.29	1.41E-12
rcp:RCAP_rcc00094	hmuV; hemin ABC transporter ATP-binding protein	P	-2.59	0.002614479
rcp:RCAP_rcc00098	hmuR; TonB-dependent hemin receptor	P	-2.03	0.000540331
rcp:RCAP_rcc00099	ABC transporter ATP-binding protein	P	-2.54	0.000310291
rcp:RCAP_rcc00100	ABC transporter ATP-binding protein	P	-2.39	5.27E-05
rcp:RCAP_rcc00101	ABC transporter permease	P	-2.56	3.04E-07
rcp:RCAP_rcc00102	ABC transporter permease	P	-2.39	3.38E-05
rcp:RCAP_rcc00103	ABC transporter periplasmic substrate-binding protein	P	-2.78	1.12E-07
rcp:RCAP_rcc00152	peptide ABC transporter periplasmic peptide-binding protein	P	-3.12	1.71E-11
rcp:RCAP_rcc00153	peptide ABC transporter permease	P	-2.04	0.01717437
rcp:RCAP_rcc00862	divalent anion:Na+ symporter	P	-2.80	5.26E-06
rcp:RCAP_rcc01032	cbiO1; cobalt ABC transporter ATP-binding protein CbiO	P	-3.63	1.58E-10
rcp:RCAP_rcc01033	cbiQ1; cobalt ABC transporter permease CbiQ	P	-2.85	7.87E-09
**rcp:RCAP_rcc01647**	**ABC transporter periplasmic substrate-binding protein**	**P**	**-9.05**	**2.63E-10**
**rcp:RCAP_rcc01648**	**ABC transporter permease**	**P**	**-2.81**	**0.021250924**
**rcp:RCAP_rcc01650**	**ABC transporter permease**	**P**	**-2.34**	**0.002481523**
**rcp:RCAP_rcc01651**	**ABC transporter ATP-binding protein**	**P**	**-2.58**	**0.032190994**
rcp:RCAP_rcc02241	tauA; taurine ABC transporter periplasmic taurine-binding protein TauA	P	-2.43	3.69E-05
**rcp:RCAP_rcc02855**	**ABC transporter permease**	**P**	**-2.52**	**0.005577702**
rcp:RCAP_rcc03015	kefC2; glutathione-regulated potassium-efflux system protein KefC	P	-2.43	3.61E-09
**rcp:RCAP_rcc03358**	**TonB-dependent receptor**	**P**	**-7.01**	**6.76E-31**
**rcp:RCAP_rcc03359**	**iron siderophore/cobalamin ABC transporter**	**P**	**-6.28**	**1.00E-15**

**rcp:RCAP_rcc03360**	**iron siderophore/cobalamin ABC transporter permease**	**P**	**-3.43**	**7.42E-07**
rcp:RCAP_rcc03361	iron siderophore/cobalamin ABC transporter ATP-binding protein	P	-3.13	0.000436387
**rcp:RCAP_rcc00021**	**cytochrome c peroxidase**	**P**	**2.27**	**3.18E-08**
rcp:RCAP_rcc00562	modA1; molybdenum ABC transporter periplasmic molybdenum-binding pr	P	2.08	2.06E-10
rcp:RCAP_rcc00904	major facilitator superfamily protein	P	2.61	4.34E-14
**rcp:RCAP_rcc01028**	**iron siderophore/cobalamin ABC transporter periplasmic iron siderophor**	**P**	**5.75**	**1.77E-27**
rcp:RCAP_rcc01029	iron siderophore/cobalamin ABC transporter permease	P	3.46	0.005689169
rcp:RCAP_rcc01030	iron siderophore/cobalamin ABC transporter permease	P	3.25	0.030348718
rcp:RCAP_rcc01557	rhodanese domain-containing protein	P	2.17	1.49E-05
rcp:RCAP_rcc02286	divalent ion symporter family	P	2.15	0.006038118
**rcp:RCAP_rcc02311**	**sodB; superoxide dismutase**	**P**	**3.08**	**9.52E-18**
**rcp:RCAP_rcc02659**	**monosacharide ABC transporter ATP-binding protein**	**P**	**4.78**	**5.11E-63**
**rcp:RCAP_rcc03065**	**efeU; ferrous iron permease EfeU**	**P**	**2.27**	**8.98E-06**
**rcp:RCAP_rcc03066**	**dyp-type peroxidase**	**P**	**2.80**	**4.89E-15**
**rcp:RCAP_rcc03067**	**EfeO; iron uptake system component**	**P**	**2.66**	**4.17E-14**
rcp:RCAP_rcc03351	rieske (2Fe-2S) domain-containing protein	P	3.00	8.89E-14
rcp:RCAP_rcp00081	rhodanese domain-containing protein	P	3.00	2.60E-05

COG Q: Secondary metabolites biosythesis, transport and catabolism

**rcp:RCAP_rcc02016**	**fumarylacetoacetate hydrolase**	**Q**	**-14.30**	**4.54E-131**
rcp:RCAP_rcc03378	CDA peptide synthetase III	Q	-3.89	0.024054772

COG R/S: General function prediction only/function unknown

rcp:RCAP_rcc00197	comF; competence protein F	R	-8.07	3.99E-14
**rcp:RCAP_rcc00383**	**FAD dependent oxidoreductase**	**R**	**-3.62**	**4.03E-28**
rcp:RCAP_rcc00417	hypothetical protein	R	-2.21	9.52E-06
rcp:RCAP_rcc00555	hypothetical protein	R	-2.11	3.50E-06
**rcp:RCAP_rcc01406**	**phage lysozyme**	**R**	**-2.25**	**0.005885493**
**rcp:RCAP_rcc01645**	**lipoprotein**	**R**	**-4.78**	**2.68E-06**
rcp:RCAP_rcc01683	terminase-like family protein	R	-2.23	0.000162269
rcp:RCAP_rcc01684	HK97 family phage portal protein	R	-12.47	5.45E-16
rcp:RCAP_rcc01686	phage prohead protease	R	-8.90	1.92E-08
rcp:RCAP_rcc01687	HK97 family phage major capsid protein	R	-11.59	1.44E-11
rcp:RCAP_rcc01691	TP901-1 family phage major tail protein	R	-12.59	1.68E-14
rcp:RCAP_rcc02077	SET domain-containing protein	R	-3.12	8.50E-27
rcp:RCAP_rcc00059	hypothetical protein	S	-3.61	8.07E-19
rcp:RCAP_rcc00087	hypothetical protein	S	-2.23	0.000120745
rcp:RCAP_rcc00093	hypothetical protein	S	-2.88	0.00341627
rcp:RCAP_rcc00142	hypothetical protein	S	-5.48	1.31E-63
rcp:RCAP_rcc00482	hypothetical protein	S	-4.00	6.78E-11
rcp:RCAP_rcc00486	hypothetical protein	S	-2.94	2.05E-27
**rcp:RCAP_rcc00542**	**hypothetical protein**	**S**	**-3.55**	**2.66E-14**
rcp:RCAP_rcc00844	hypothetical protein	S	-4.64	1.50E-27
rcp:RCAP_rcc00845	hypothetical protein	S	-3.56	5.51E-26
rcp:RCAP_rcc01039	hypothetical protein	S	-2.19	0.027191181
rcp:RCAP_rcc01052	hypothetical protein	S	-3.33	0.01861444
rcp:RCAP_rcc01055	hypothetical protein	S	-5.12	2.98E-09
rcp:RCAP_rcc01061	hypothetical protein	S	-2.14	0.009672288
rcp:RCAP_rcc01064	hypothetical protein	S	-5.23	1.59E-27
rcp:RCAP_rcc01065	hypothetical protein	S	-5.22	4.22E-13
rcp:RCAP_rcc01066	pyp; photoactive yellow protein	S	-5.31	3.69E-34
rcp:RCAP_rcc01067	hypothetical protein	S	-5.84	1.76E-40
rcp:RCAP_rcc01070	hypothetical protein	S	-5.36	1.33E-07
rcp:RCAP_rcc01071	hypothetical protein	S	-4.13	6.90E-06
rcp:RCAP_rcc01074	hypothetical protein	S	-5.65	0.002454789

rcp:RCAP_rcc01076	hypothetical protein	S	-9.85	5.15E-33
rcp:RCAP_rcc01080	hypothetical protein	S	-6.89	2.06E-12
rcp:RCAP_rcc01221	hypothetical protein	S	-3.46	4.67E-10
**rcp:RCAP_rcc01232**	**hypothetical protein**	**S**	**-68.61**	**6.61E-61**
**rcp:RCAP_rcc01233**	**hypothetical protein**	**S**	**-6.54**	**3.65E-05**
rcp:RCAP_rcc01350	hypothetical protein	S	-9.34	5.99E-58
rcp:RCAP_rcc01351	hypothetical protein	S	-4.03	5.85E-07
rcp:RCAP_rcc01405	hypothetical protein	S	-2.00	1.71E-06
**rcp:RCAP_rcc01408**	**hypothetical protein**	**S**	**-2.29**	**0.037248247**
rcp:RCAP_rcc01413	hypothetical protein	S	-2.38	0.002832592
rcp:RCAP_rcc01424	hypothetical protein	S	-2.91	0.034005902
rcp:RCAP_rcc01761	hypothetical protein	S	-11.31	1.83E-104
rcp:RCAP_rcc01918	hypothetical protein	S	-7.29	0.002668716
rcp:RCAP_rcc01933	hypothetical protein	S	-2.49	3.11E-06
rcp:RCAP_rcc02002	hypothetical protein	S	-2.70	0.000251135
rcp:RCAP_rcc02068	ice nucleation protein repeat family protein	S	-13.06	4.01E-100
rcp:RCAP_rcc02078	hypothetical protein	S	-3.04	1.03E-11
rcp:RCAP_rcc02297	GNAT family acetyltransferase	S	-2.44	1.82E-12
rcp:RCAP_rcc02298	GNAT family acetyltransferase	S	-2.19	3.45E-10
rcp:RCAP_rcc02395	hypothetical protein	S	-2.59	2.70E-09
rcp:RCAP_rcc02415	hypothetical protein	S	-6.51	2.10E-39
**rcp:RCAP_rcc02463**	**hemolysin-type calcium-binding repeat family protein**	**S**	**-2.84**	**1.51E-11**
rcp:RCAP_rcc02488	hypothetical protein	S	-6.22	5.61E-35
rcp:RCAP_rcc02597	hypothetical protein	S	-3.40	1.91E-25
rcp:RCAP_rcc02600	hypothetical protein	S	-2.70	6.54E-11
rcp:RCAP_rcc02630	heme NO binding domain-containing protein	S	-4.94	1.11E-22
rcp:RCAP_rcc02708	hypothetical protein	S	-2.19	3.82E-16
**rcp:RCAP_rcc02815**	**lipoprotein**	**S**	**-4.72**	**1.09E-21**
rcp:RCAP_rcc02888	hypothetical protein	S	-2.50	0.00545617
rcp:RCAP_rcc02980	hypothetical protein	S	-4.70	1.28E-27
rcp:RCAP_rcc03000	hypothetical protein	S	-3.57	1.10E-33
rcp:RCAP_rcc03052	hypothetical protein	S	-2.61	1.74E-14
rcp:RCAP_rcc03116	hypothetical protein	S	-2.92	0.0018565
rcp:RCAP_rcc03121	hypothetical protein	S	-2.48	0.007700904
rcp:RCAP_rcc03299	hypothetical protein	S	-6.83	2.60E-23
**rcp:RCAP_rcc03339**	**hypothetical protein**	**S**	**-3.38**	**7.15E-05**
**rcp:RCAP_rcc03340**	**hypothetical protein**	**S**	**-4.44**	**6.21E-10**
**rcp:RCAP_rcc03341**	**hypothetical protein**	**S**	**-4.47**	**4.84E-13**
rcp:RCAP_rcc03356	hypothetical protein	S	-2.82	2.37E-05
**rcp:RCAP_rcc03377**	**hypothetical protein**	**S**	**-8.55**	**1.02E-62**
rcp:RCAP_rcc03405	hypothetical protein	S	-2.38	8.69E-05
rcp:RCAP_rcc03484	hypothetical protein	S	-7.65	9.83E-38
rcp:RCAP_rcc03526	hypothetical protein	S	-6.13	3.47E-71
rcp:RCAP_rcc03528	hypothetical protein	S	-9.23	1.22E-117
rcp:RCAP_rcp00010	hypothetical protein	S	-6.01	0.013833966
rcp:RCAP_rcc00171	hypothetical protein	S	-3.16	1.71E-06
rcp:RCAP_rcc00180	Hpt domain-containing protein	S	-3.65	4.60E-22
rcp:RCAP_rcc00216	hypothetical protein	S	-4.20	1.77E-19
rcp:RCAP_rcc00353	hypothetical protein	S	-2.70	1.66E-17
rcp:RCAP_rcc00630	ice nucleation protein repeat family protein	S	-12.22	1.68E-81
rcp:RCAP_rcc01041	hypothetical protein	S	-2.07	0.000530382
rcp:RCAP_rcc01079	hypothetical protein	S	-5.41	1.13E-09
rcp:RCAP_rcc01138	hypothetical protein	S	-7.30	5.96E-28
rcp:RCAP_rcc01139	hypothetical protein	S	-6.14	1.58E-35
rcp:RCAP_rcc01242	hemolysin-type calcium-binding repeat family protein	S	-5.25	7.89E-25
**rcp:RCAP_rcc01409**	**hypothetical protein**	**S**	**-3.00**	**5.77E-07**
rcp:RCAP_rcc01425	hypothetical protein	S	-2.31	0.000125114

rcp:RCAP_rcc01507	hypothetical protein	S	-4.03	7.37E-26
**rcp:RCAP_rcc01652**	**hypothetical protein**	**S**	**-7.75**	**3.66E-09**
**rcp:RCAP_rcc01653**	**YVTN beta-propeller repeat family protein**	**S**	**-5.13**	**7.27E-10**
**rcp:RCAP_rcc01654**	**hypothetical protein**	**S**	**-4.40**	**4.78E-05**
**rcp:RCAP_rcc01655**	**hypothetical protein**	**S**	**-4.60**	**0.000216247**
rcp:RCAP_rcc01662	hypothetical protein	S	-3.71	2.46E-24
rcp:RCAP_rcc01685	hypothetical protein	S	-7.54	0.000114304
rcp:RCAP_rcc01688	hypothetical protein	S	-22.36	1.77E-19
rcp:RCAP_rcc01690	hypothetical protein	S	-10.17	2.68E-07
rcp:RCAP_rcc01694	hypothetical protein	S	-8.06	0.000297794
rcp:RCAP_rcc01695	hypothetical protein	S	-14.65	8.73E-15
rcp:RCAP_rcc01696	hypothetical protein	S	-8.67	4.93E-11
rcp:RCAP_rcc01698	hypothetical protein	S	-5.80	4.50E-16
rcp:RCAP_rcc01865	hypothetical protein	S	-7.78	3.21E-14
rcp:RCAP_rcc01917	hypothetical protein	S	-5.79	0.000359903
rcp:RCAP_rcc02063	M10 family peptidase	S	-12.58	1.88E-67
rcp:RCAP_rcc02067	type 11 family methyltransferase	S	-19.82	3.08E-116
rcp:RCAP_rcc02152	TM2 domain-containing protein	S	-2.26	8.13E-08
rcp:RCAP_rcc02172	hypothetical protein	S	-4.82	1.74E-12
**rcp:RCAP_rcc02390**	**alkane 1-monooxygenase**	**S**	**-37.10**	**1.02E-95**
rcp:RCAP_rcc02591	surface presentation of antigens protein family	S	-11.08	9.51E-80
rcp:RCAP_rcc02610	hypothetical protein	S	-10.38	3.65E-58
rcp:RCAP_rcc02623	hypothetical protein	S	-8.21	5.06E-09
rcp:RCAP_rcc02706	hypothetical protein	S	-3.26	1.47E-05
rcp:RCAP_rcc03122	FRG domain-containing protein	S	-5.60	3.19E-32
rcp:RCAP_rcc03209	hypothetical protein	S	-2.14	1.13E-17
rcp:RCAP_rcc03485	hypothetical protein	S	-10.78	1.39E-117
rcp:RCAP_rcc03487	hypothetical protein	S	-10.05	3.77E-115
rcp:RCAP_rcc00635	von Willebrand factor type A domain-containing protein	R	2.09	3.34E-07
rcp:RCAP_rcc00980	phage virion morphogenesis protein	R	2.06	0.000158896
**rcp:RCAP_rcc02118**	**cat; chloramphenicol acetyltransferase**	**R**	**3.20**	**5.52E-22**
rcp:RCAP_rcc02554	NmrA family protein	R	2.16	3.24E-05
rcp:RCAP_rcc02683	type 11 family methyltransferase	R	2.29	1.81E-06
rcp:RCAP_rcc03419	LrgA family protein	R	2.34	2.98E-08
rcp:RCAP_rcc00203	hypothetical protein	S	2.14	0.01490823
rcp:RCAP_rcc00283	hypothetical protein	S	3.26	1.65E-14
rcp:RCAP_rcc00575	hypothetical protein	S	2.06	7.60E-08
**rcp:RCAP_rcc00642**	**peptidoglycan binding domain-containing protein**	**S**	**2.30**	**1.40E-11**
rcp:RCAP_rcc00734	lipoprotein	S	2.01	7.82E-07
rcp:RCAP_rcc00737	hypothetical protein	S	7.98	1.49E-36
**rcp:RCAP_rcc00891**	**hypothetical protein**	**S**	**5.99**	**1.07E-27**
rcp:RCAP_rcc00968	hypothetical protein	S	2.87	1.46E-12
rcp:RCAP_rcc00969	hypothetical protein	S	3.52	3.55E-05
rcp:RCAP_rcc00970	hypothetical protein	S	5.90	7.01E-18
rcp:RCAP_rcc00971	hypothetical protein	S	3.16	2.75E-14
rcp:RCAP_rcc00972	hypothetical protein	S	5.01	0.000556931
rcp:RCAP_rcc00973	hypothetical protein	S	4.77	1.30E-09
rcp:RCAP_rcc00976	hypothetical protein	S	5.14	3.57E-20
rcp:RCAP_rcc00983	hypothetical protein	S	2.55	0.009672288
rcp:RCAP_rcc00987	hypothetical protein	S	4.11	5.02E-07
rcp:RCAP_rcc01528	hypothetical protein	S	2.08	1.19E-09
rcp:RCAP_rcc01613	hypothetical protein	S	2.91	1.51E-25
rcp:RCAP_rcc01738	katG; catalase/peroxidase	S	2.79	2.26E-07
rcp:RCAP_rcc01776	trimethylamine methyltransferase	S	2.32	2.38E-15
rcp:RCAP_rcc01897	hypothetical protein	S	7.30	1.38E-16
rcp:RCAP_rcc01940	hemolysin-type calcium-binding repeat family protein	S	3.22	7.14E-24
rcp:RCAP_rcc02074	ferredoxin domain-containing protein	S	2.50	1.72E-13

**rcp:RCAP_rcc02119**	**type 12 family methyltransferase**	**S**	**2.47**	**5.91E-17**
rcp:RCAP_rcc02161	hypothetical protein	S	3.05	3.80E-10
rcp:RCAP_rcc02190	zntA2; heavy metal translocating P-type ATPase	S	3.69	2.94E-11
rcp:RCAP_rcc02195	HNH endonuclease	S	5.06	1.94E-30
rcp:RCAP_rcc02273	hypothetical protein	S	13.97	1.49E-31
rcp:RCAP_rcc02283	hypothetical protein	S	2.82	0.040266947
rcp:RCAP_rcc02285	hypothetical protein	S	3.05	0.002448544
rcp:RCAP_rcc02288	hypothetical protein	S	2.03	0.000240689
rcp:RCAP_rcc02290	hypothetical protein	S	2.21	0.000539225
**rcp:RCAP_rcc02293**	**hypothetical protein**	**S**	**2.01**	**0.008677116**
rcp:RCAP_rcc02321	hypothetical protein	S	3.22	9.00E-11
rcp:RCAP_rcc02479	lipoprotein	S	2.90	1.07E-05
**rcp:RCAP_rcc02646**	**hypothetical protein**	**S**	**2.01**	**0.00088028**
rcp:RCAP_rcc02665	hemolysin-type calcium-binding repeat family protein	S	3.04	4.35E-08
rcp:RCAP_rcc02726	hypothetical protein	S	2.83	0.001409684
rcp:RCAP_rcc02764	hypothetical protein	S	11.59	1.29E-69
rcp:RCAP_rcc02929	hypothetical protein	S	2.20	9.14E-10
rcp:RCAP_rcc02947	hypothetical protein	S	2.48	7.89E-10
rcp:RCAP_rcc02948	hypothetical protein	S	2.42	1.28E-05
rcp:RCAP_rcc02949	hypothetical protein	S	2.51	1.15E-10
rcp:RCAP_rcc03213	hypothetical protein	S	2.00	8.17E-11
**rcp:RCAP_rcc03403**	**hypothetical protein**	**S**	**5.50**	**2.05E-16**
**rcp:RCAP_rcc03429**	**hypothetical protein**	**S**	**5.20**	**2.26E-06**
rcp:RCAP_rcp00067	hypothetical protein	S	10.39	2.30E-14
**rcp:RCAP_rcp00069**	**hypothetical protein**	**S**	**9.28**	**4.93E-16**
**rcp:RCAP_rcp00077**	**hypothetical protein**	**S**	**6.49**	**0.000171515**
rcp:RCAP_rcp00083	hypothetical protein	S	2.09	0.000934249
**rcp:RCAP_rcc00104**	**hypothetical protein**	**S**	**2.02**	**0.000327421**
**rcp:RCAP_rcc00423**	**hypothetical protein**	**S**	**6.28**	**4.96E-18**
rcp:RCAP_rcc00424	hypothetical protein	S	4.15	6.54E-14
rcp:RCAP_rcc00697	hypothetical protein	S	2.30	7.51E-07
rcp:RCAP_rcc00747	hypothetical protein	S	2.52	2.89E-13
rcp:RCAP_rcc00880	CHAP domain-containing protein	S	2.06	7.14E-12
**rcp:RCAP_rcc00885**	**hypothetical protein**	**S**	**3.88**	**1.97E-10**
rcp:RCAP_rcc00888	hypothetical protein	S	5.55	3.95E-10
rcp:RCAP_rcc00889	NosL family protein	S	7.27	1.39E-35
**rcp:RCAP_rcc00890**	**hypothetical protein**	**S**	**5.94**	**7.28E-23**
rcp:RCAP_rcc00892	hypothetical protein	S	2.44	1.00E-08
rcp:RCAP_rcc00901	hypothetical protein	S	2.75	1.98E-06
rcp:RCAP_rcc00964	phage tail fiber protein	S	2.22	1.47E-08
rcp:RCAP_rcc00965	phage tail assembly protein	S	4.01	6.59E-05
rcp:RCAP_rcc00966	hypothetical protein	S	3.44	0.001041814
rcp:RCAP_rcc00967	hypothetical protein	S	3.67	0.022736002
rcp:RCAP_rcc00974	hypothetical protein	S	5.80	2.09E-06
rcp:RCAP_rcc00975	cyclic nucleotide-binding domain-containing protein	S	3.35	1.39E-06
rcp:RCAP_rcc00977	hypothetical protein	S	4.73	2.21E-23
rcp:RCAP_rcc00978	hypothetical protein	S	4.70	4.30E-14
rcp:RCAP_rcc00979	hypothetical protein	S	5.09	3.75E-31
rcp:RCAP_rcc00982	hypothetical protein	S	2.34	5.17E-06
rcp:RCAP_rcc00985	hypothetical protein	S	3.06	7.91E-09
rcp:RCAP_rcc00986	hypothetical protein	S	4.08	2.79E-14
rcp:RCAP_rcc00988	hypothetical protein	S	4.52	1.46E-10
rcp:RCAP_rcc00989	lysozyme	S	4.08	1.39E-16
rcp:RCAP_rcc01019	PHP domain-containing protein	S	2.08	0.014741388
**rcp:RCAP_rcc01027**	**hypothetical protein**	**S**	**11.07**	**1.48E-37**
**rcp:RCAP_rcc01035**	**hypothetical protein**	**S**	**2.01**	**1.49E-06**
rcp:RCAP_rcc01115	lipoprotein	S	2.12	8.44E-14

rcp:RCAP_rcc01155	hypothetical protein	S	2.56	1.80E-07
rcp:RCAP_rcc01184	CsbD family protein	S	2.26	8.48E-07
rcp:RCAP_rcc01319	hypothetical protein	S	2.74	5.56E-05
**rcp:RCAP_rcc01423**	**hypothetical protein**	**S**	**2.55**	**1.70E-16**
rcp:RCAP_rcc01589	ErfK/YbiS/YcfS/YnhG family protein	S	2.08	3.20E-05
**rcp:RCAP_rcc01721**	**TfoX domain-containing protein**	**S**	**2.42**	**5.30E-07**
rcp:RCAP_rcc01890	hypothetical protein	S	2.36	8.60E-05
rcp:RCAP_rcc01900	hemolysin-type calcium-binding repeat family protein	S	3.16	3.90E-37
**rcp:RCAP_rcc02141**	**hypothetical protein**	**S**	**8.07**	**1.07E-23**
rcp:RCAP_rcc02189	hypothetical protein	S	2.05	3.41E-07
rcp:RCAP_rcc02282	hypothetical protein	S	3.54	0.012279263
rcp:RCAP_rcc02333	hypothetical protein	S	2.14	3.04E-06
**rcp:RCAP_rcc02454**	**PAS domain-containing protein**	**S**	**5.34**	**1.27E-09**
rcp:RCAP_rcc02511	hypothetical protein	S	2.06	7.06E-12
rcp:RCAP_rcc02520	integrin alpha repeat/hemolysin-type calcium-binding repeat family protein	S	2.86	1.78E-13
**rcp:RCAP_rcc02658**	**lipoprotein**	**S**	**4.41**	**3.30E-52**
rcp:RCAP_rcc02684	polyphosphate kinase 2 domain-containing protein	S	4.06	3.94E-13
rcp:RCAP_rcc02727	hypothetical protein	S	2.10	0.001330574
rcp:RCAP_rcc02728	ATPase AAA	S	2.11	0.000679583
rcp:RCAP_rcc02844	hypothetical protein	S	16.20	3.73E-22
rcp:RCAP_rcc03057	hypothetical protein	S	2.14	2.47E-07
rcp:RCAP_rcc03163	hypothetical protein	S	2.14	3.15E-14
rcp:RCAP_rcc03181	hypothetical protein	S	2.87	1.35E-29
rcp:RCAP_rcc03214	hypothetical protein	S	2.24	0.000125925
rcp:RCAP_rcc03466	hypothetical protein	S	2.67	0.000117827
**rcp:RCAP_rcp00068**	**M4 family peptidase**	**S**	**3.43**	**7.35E-12**

COG T: Signal transduction mechanisms

rcp:RCAP_rcc00042	PAS/PAC sensor domain-containing protein	T	-7.02	1.39E-49
**rcp:RCAP_rcc00045**	**regA1; photosynthetic apparatus regulatory protein RegA**	**T**	**-73.77**	**1.15E-228**
rcp:RCAP_rcc00181	response regulator receiver domain/protein phosphatase 2C domain-conta	T	-12.65	1.77E-63
rcp:RCAP_rcc00346	diguanylate cyclase/phosphodiesterase	T	-3.44	2.67E-28
**rcp:RCAP_rcc00356**	**cyclic nucleotide-binding domain-/cystathionine beta-synthase domain**	**T**	**-3.30**	**2.26E-15**
rcp:RCAP_rcc00537	response regulator receiver protein	T	-10.16	1.93E-84
**rcp:RCAP_rcc00567**	**nifA1; Nif-specific regulatory protein**	**T**	**-5.30**	**3.77E-12**
rcp:RCAP_rcc00620	response regulator receiver modulated diguanylate cyclase/phosphodieste	T	-11.25	1.44E-92
rcp:RCAP_rcc00621	signal transduction histidine kinase	T	-3.95	6.49E-31
**rcp:RCAP_rcc01020**	**diguanylate cyclase/phosphodiesterase**	**T**	**-6.19**	**1.39E-42**
rcp:RCAP_rcc01393	LuxR family two component transcriptional regulator	T	-2.06	0.036901856
rcp:RCAP_rcc02070	ArsR family transcriptional regulator/protein tyrosine phosphatase	T	-8.25	0.004036144
rcp:RCAP_rcc02075	PAS/PAC sensor domain-containing protein	T	-3.93	2.26E-17
rcp:RCAP_rcc02539	diguanylate cyclase/phosphodiesterase	T	-7.68	4.53E-40
rcp:RCAP_rcc02629	diguanylate cyclase/phosphodiesterase	T	-5.00	2.49E-24
**rcp:RCAP_rcc02856**	**PAS/PAC sensor domain-containing protein**	**T**	**-34.31**	**2.25E-118**
**rcp:RCAP_rcc02857**	**diguanylate cyclase/phosphodiesterase**	**T**	**-12.81**	**1.40E-103**
rcp:RCAP_rcc03176	PAS/PAC sensor domain-containing protein	T	-3.99	4.72E-55
rcp:RCAP_rcc03177	EAL domain-containing protein	T	-9.02	4.52E-59
rcp:RCAP_rcc03301	diguanylate cyclase/phosphodiesterase	T	-3.37	2.09E-25
rcp:RCAP_rcc03323	rsbV; anti-sigma-factor antagonist	T	-22.08	2.09E-89
rcp:RCAP_rcc03324	rsbW; anti-sigma regulatory factor	T	-3.46	1.32E-33
rcp:RCAP_rcc03452	sensor histidine kinase/response regulator receiver protein	T	-11.80	8.22E-64
rcp:RCAP_rcp00117	EAL domain-containing protein	T	-4.33	1.03E-35
rcp:RCAP_rcp00137	PAS/PAC sensor domain-containing protein	T	-9.88	1.84E-67
rcp:RCAP_rcc02076	diguanylate cyclase/phosphodiesterase	T	-2.02	5.87E-11
**rcp:RCAP_rcc00643**	**diguanylate cyclase/phosphodiesterase**	**T**	**3.30**	**1.26E-17**
rcp:RCAP_rcc00711	universal stress family protein	T	3.10	5.99E-13

rcp:RCAP_rcc00778	hupR; hydrogenase transcriptional regulator HupR	T	3.17	1.13E-26
rcp:RCAP_rcc00783	diguanylate cyclase/phosphodiesterase	T	2.09	1.62E-10
rcp:RCAP_rcc01110	diguanylate cyclase/phosphodiesterase	T	2.82	4.91E-15
rcp:RCAP_rcc01116	PhoH family protein	T	2.29	1.60E-13
**rcp:RCAP_rcc01156**	**UspA domain-containing protein**	**T**	**14.15**	**2.22E-52**
**rcp:RCAP_rcc01495**	**fusA2; translation elongation factor G**	**T**	**7.00**	**1.44E-08**
rcp:RCAP_rcc02197	two component AraC family transcriptional regulator	T	7.71	1.04E-20
rcp:RCAP_rcc02198	histidine kinase	T	5.64	6.30E-06
rcp:RCAP_rcc02289	two-component response regulator receiver protein	T	3.05	6.39E-10
rcp:RCAP_rcc02292	signal transduction histidine kinase	T	2.39	4.40E-07
**rcp:RCAP_rcc02294**	**signal transduction histidine kinase**	**T**	**2.73**	**1.22E-14**
rcp:RCAP_rcc02590	dksA2; DnaK suppressor protein	T	2.86	7.80E-09
rcp:RCAP_rcc02849	dorS; DMSO/TMAO-sensor hybrid histidine kinase	T	2.92	2.97E-06
rcp:RCAP_rcp00066	NnrS family protein	T	6.81	3.90E-16

COG U: Intracellular trafficking, secretion, and vesicular transport

rcp:RCAP_rcc02066	hemolysin D	U	-8.85	2.02E-26

COG V: Defense mechanisms

rcp:RCAP_rcc00615	acrA; acriflavine resistance protein A	V	-2.13	1.63E-08
rcp:RCAP_rcc00616	acrB; acriflavine resistance protein B	V	-2.09	1.29E-12
rcp:RCAP_rcc02065	secretion ATP-binding protein, HlyB family	V	-18.81	1.20E-139
rcp:RCAP_rcc00633	ABC transporter permease	V	2.25	0.000256044
**rcp:RCAP_rcc00886**	**ABC transporter ATP-binding protein**	**V**	**6.19**	**2.53E-14**

Table S1: WT-ΔregA DEGs under photosynthetic conditions in PY medium organized by COG and regulation strength. Genes highlighted in red indicate repression by RegA, genes highlighted in green indicate activation by RegA. Genes highlighted in bold-face type indicate that they are regulated in both PY and RCV medium.

Table S2: WT-Δ*regA* DEGs under photosynthetic conditions in RCV medium

Gene		COG	FC	p-val
COG C: Energy production and storage				
**rcc02015**	**aldH1; aldehyde dehydrogenase**	**C**	**-6.88**	**3.31E-142**
**rcc02530**	**pucB; light-harvesting protein B-800/850 subunit beta**	**C**	**-5.34**	**5.33E-122**
**rcc01646**	**cytochrome c domain-containing protein**	**C**	**-4.11**	**1.19E-22**
**rcc02532**	**pucC2; protein PucC**	**C**	**-3.78**	**8.88E-106**
**rcc02531**	**pucA; light-harvesting protein B-800/850 subunit alpha**	**C**	**-3.59**	**1.83E-65**
**rcc02533**	**pucDE; light-harvesting protein B-800/850 subunit gamm**	**C**	**-3.30**	**6.52E-57**
**rcc01656**	**cycA2; cytochrome c2**	**C**	**-3.02**	**6.84E-10**
**rcc02702**	**cytochrome c/b561 family protein**	**C**	**-2.94**	**1.17E-37**
**rcc00691**	**pufB; light-harvesting protein B-870 subunit beta**	**C**	**-2.41**	**1.54E-32**
**rcc00693**	**pufL; photosynthetic reaction center subunit L**	**C**	**-2.33**	**1.54E-39**
**rcc00694**	**pufM; photosynthetic reaction center subunit M**	**C**	**-2.33**	**3.91E-38**
**rcc00692**	**pufA; light-harvesting protein B-870 subunit alpha**	**C**	**-2.32**	**6.10E-38**
rcc03085	cydA; cytochrome d ubiquinol oxidase subunit I	C	-2.30	3.26E-24
**rcc00690**	**pufQ; cytochrome, subunit PufQ**	**C**	**-2.23**	**1.67E-29**
rcc03007	cysJ; sulfite reductase (NADPH) flavoprotein subunit alpha	C	-2.17	1.33E-15
rcc03084	cydB; cytochrome d ubiquinol oxidase subunit II	C	-2.12	1.27E-18
rcc01174	D-2-hydroxyglutarate dehydrogenase	C	-2.02	1.07E-12
**rcc03016**	**draT; NAD(+)--dinitrogen-reductase ADP-D-ribosyltransfe**	**C**	**-2.05**	**1.21E-08**
**rcc01163**	**ccoI; cbb3-type cytochrome c oxidase biogenesis protein**	**C**	**2.04**	**1.82E-24**
**rcc00762**	**hoxW; hydrogenase maturation factor**	**C**	**2.05**	**4.45E-06**
**rcc01162**	**ccoH; cbb3-type cytochrome c oxidase biogenesis protei**	**C**	**2.19**	**1.37E-15**
**rcc03037**	**fdhC; NAD-dependent formate dehydrogenase subunit g**	**C**	**2.28**	**6.46E-17**
**rcc00022**	**aldo/keto reductase family oxidoreductase**	**C**	**2.52**	**2.24E-47**
**rcc03035**	**fdhA; NAD-dependent formate dehydrogenase subunit a**	**C**	**2.63**	**3.87E-44**
**rcc03036**	**fdhB; NAD-dependent formate dehydrogenase subunit b**	**C**	**2.75**	**3.04E-35**
**rcc01161**	**ccoG; cbb3-type cytochrome c oxidase accessory protein**	**C**	**3.34**	**2.89E-81**
**rcc01728**	**nifJ; pyruvate-flavodoxin oxidoreductase**	**C**	**3.67**	**1.60E-70**
rcc02448	aldH2; aldehyde dehydrogenase	C	3.96	7.40E-50
**rcp00072**	**nosY; cooper ABC transporter permease**	**C**	**6.33**	**7.98E-47**
**rcc00767**	**hupA; hydrogenase small subunit**	**C**	**7.64**	**2.33E-171**
**rcc00769**	**hupC; hydrogenase, cytochrome b subunit**	**C**	**7.79**	**3.39E-101**
**rcc00768**	**hupB; hydrogenase large subunit**	**C**	**7.94**	**7.49E-168**
**rcc01157**	**ccoN; cbb3-type cytochrome c oxidase subunit I**	**C**	**8.39**	**2.23E-232**
**rcc01160**	**ccoP; cbb3-type cytochrome c oxidase subunit III**	**C**	**9.00**	**1.92E-250**
rcp00070	nosX; NosX protein	C	9.28	3.71E-72
**rcc01158**	**ccoO; cbb3-type cytochrome c oxidase subunit II**	**C**	**9.66**	**4.48E-239**
**rcp00074**	**nosD; nitrous oxide maturation protein**	**C**	**9.90**	**3.43E-114**
**rcp00073**	**nosF; copper ABC transporter ATP-binding protein**	**C**	**10.02**	**6.88E-46**
**rcc01159**	**ccoQ; cbb3-type cytochrome c oxidase subunit IV**	**C**	**10.07**	**2.34E-230**
**rcp00075**	**nosZ; nitrous-oxide reductase**	**C**	**13.15**	**3.10E-216**

rcp00071	nosL; nitrous oxide reductase accessory protein	C	13.73	9.72E-78

COG E: Amino acid transport and metabolism

**rcc00516**	**tpl; tyrosine phenol-lyase**	**E**	**-9.65**	**1.01E-173**
rcc01226	urtB; urea ABC transporter urea binding protein	E	-2.73	5.69E-12
rcc02183	potG2; polyamine ABC transporter ATP-binding protein	E	2.01	1.03E-08
rcc02182	class III aminotransferase	E	2.04	2.12E-20
rcc02185	potI3; polyamine ABC transporter permease	E	2.13	3.01E-20
rcp00003	polar amino acid ABC transporter	E	2.25	4.77E-28
rcc02186	potF; polyamine ABC transporter periplasmic polyamine-b	E	2.31	2.17E-36
rcc03161	dat; D-amino-acid transaminase	E	2.56	1.37E-51
rcc02449	potC3; polyamine ABC transporter permease	E	2.96	2.06E-15
rcc03146	gabT1; 4-aminobutyrate aminotransferase	E	2.96	4.84E-41
rcc02450	potB4; polyamine ABC transporter permease	E	3.01	4.67E-18
**rcc02452**	**potD4; polyamine ABC transporter periplasmic polyamin**	**E**	**4.28**	**1.81E-75**
**rcc02451**	**potA4; polyamine ABC transporter ATP-binding protein**	**E**	**4.53**	**2.75E-22**
**rcc00484**	**ald; alanine dehydrogenase**	**E**	**60.53**	**0**

COG F: Nucleotide transport and metabolism

**rcc03092**	**apt; adenine phosphoribosyltransferase**	**F**	**-2.65**	**5.35E-34**

COG G: Carbohydrate transport and metabolism

**rcc03013**	**DeoC/LacD family aldolase**	**G**	**-4.77**	**2.86E-55**
**rcc01657**	**exaA2; quinoprotein ethanol dehydrogenase**	**G**	**-2.88**	**3.25E-41**
rcc01834	fbp; fructose-bisphosphatase	G	-2.15	5.86E-32
rcc00420	pykA1; pyruvate kinase	G	-2.10	8.88E-12
**rcc02660**	**monosacharide ABC transporter permease**	**G**	**2.46**	**1.01E-31**
**rcc02657**	**monosacharide ABC transporter**	**G**	**2.58**	**2.67E-58**

COG GRP: Glycyl radical enzyme microcompartment

**rcc02208**	**pduB; propanediol utilization protein PduB**	**GRP**	**2.01**	**0.006908136**

COG H: Coenzyme transport and metabolism

**rcc02430**	**nahG; salicylate hydroxylase**	**H,C**	**-3.07**	**2.35E-51**
**rcc03091**	**mtnP; S-methyl-5-thioadenosine phosphorylase**	**H**	**-2.86**	**1.13E-44**
**rcc01172**	**hemE; uroporphyrinogen decarboxylase**	**H**	**-2.68**	**4.18E-55**
**rcc01235**	**pyrimidine 5'-nucleotidase**	**H**	**-2.48**	**1.74E-06**
**rcc01644**	**FMN-binding domain-containing protein**	**H,C,K**	**-2.32**	**1.28E-09**
**rcc01447**	**hemA; 5-aminolevulinate synthase**	**H**	**-2.04**	**1.83E-37**
**rcc00028**	**idi1; isopentenyl-diphosphate delta-isomerase**	**H**	**-6.09**	**1.78E-41**
**rcc03362**	**cbiZ; adenosylcobinamide amidohydrolase**	**H**	**-2.36**	**1.93E-05**
**rcp00134**	**citG; triphosphoribosyl-dephospho-CoA synthase**	**H**	**-2.00**	**0.005194848**
**rcc02638**	**calcium-binding EF-hand domain-containing protein**	**H**	**2.05**	**3.50E-11**
rcc01034	CbiM family cobalamin biosynthesis protein	H	2.21	4.60E-22
**rcc00712**	**moaA1; molybdenum cofactor biosynthesis protein A**	**H**	**2.23**	**2.61E-28**

rcc00151	hemN1; oxygen-independent coproporphyrinogen-III oxid	H	4.44	1.77E-34

COG I: Lipid transport and metabolism

**rcc02620**	**acsA2; acetate--CoA ligase**	**I**	**-2.47**	**5.69E-45**
rcc00696	dxs1; 1-deoxy-D-xylulose-5-phosphate synthase	I,H	-2.00	9.74E-32
rcc02992	atoB1; acetyl-CoA acetyltransferase	I	2.24	1.99E-12

COG J: Translation, ribosomal structure and biogenesis

rcc02664	mtnA; methylthioribose-1-phosphate isomerase	J	-2.54	3.75E-31

COG K: Transcription

**rcc01722**	**BadM/Rrf2 family transcriptional regulator**	**K**	**-4.25**	**8.96E-44**
**rcc00568**	**rpoN; RNA polymerase sigma-54 factor**	**K**	**-3.34**	**1.17E-11**
rcc03255	Crp/Fnr family transcriptional regulator	K	-2.10	2.71E-13
rcp00113	AraC family transcriptional regulator	K	2.26	3.01E-09
rcc03145	AsnC/Lrp family transcriptional regulator	K	3.80	2.57E-29
rcc00112	AraC family transcriptional regulator	K	4.19	2.57E-15
rcc01048	AraC family transcriptional regulator	K	4.88	2.09E-16
**rcp00076**	**nosR; nitrous-oxide reductase expression regulator**	**K**	**5.78**	**8.05E-78**
**rcc02453**	**Fis family sigma54 specific transcriptional regulator**	**K**	**6.04**	**8.30E-42**

COG L: Replication, recombination, and repair

rcc01274	RAMP family CRISPR-associated protein	L	-2.18	1.17E-32

COG M: Cell wall/membrane/envelope biogenesis

**rcc00887**	**hypothetical protein**	**M**	**2.89**	**1.18E-13**
rcc02377	tonB; protein TonB; K03832 periplasmic protein	M	2.66	8.21E-22

COG N: Cell motility

**rcc02151**	**methyl-accepting chemotaxis sensory transducer**	**N**	**-4.25**	**3.21E-39**
rcc01073	gvpA; gas vesicle protein GvpA	N	2.33	1.39E-18

COG O: Posttranslational modification, protein turnover, chaperones

**rcc00777**	**hypB; hydrogenase nickel incorporation protein**	**O**	**2.03**	**3.42E-20**
**rcc00780**	**hypD; hydrogenase expression/formation protein**	**O**	**2.03**	**1.66E-15**
**rcc00770**	**hupD; hydrogenase maturation protease**	**O**	**2.27**	**1.52E-17**
**rcc00774**	**hupJ; rubredoxin HupJ**	**O**	**2.46**	**2.11E-29**
**rcc00640**	**S1/S6 family peptidase**	**O**	**2.48**	**1.20E-07**
**rcc00772**	**hupG; hydrogenase expression/formation protein**	**O**	**2.68**	**7.79E-26**
**rcc00773**	**hupH; hydrogenase expression/formation protein**	**O**	**2.70**	**3.50E-39**
**rcc00775**	**hupK; hydrogenase expression/formation protein**	**O**	**2.73**	**1.86E-22**
rcc00485	gst; glutathione S-transferase	O	2.81	6.89E-61
**rcc00771**	**hupF; hydrogenase maturation chaperone**	**O**	**2.90**	**1.53E-20**
rcc02472	bsaA2; glutathione peroxidase	O	3.66	4.76E-14

COG P: Inorganic ion transport and metabolism

**rcc01647**	**ABC transporter periplasmic substrate-binding protein**	**P**	**-3.81**	**5.95E-18**
**rcc03359**	**iron siderophore/cobalamin ABC transporter**	**P**	**-3.24**	**2.87E-07**
**rcc03358**	**TonB-dependent receptor**	**P**	**-3.15**	**5.57E-07**
**rcc01651**	**ABC transporter ATP-binding protein**	**P**	**-3.01**	**8.41E-11**
**rcc03360**	**iron siderophore/cobalamin ABC transporter permease**	**P**	**-2.89**	**4.52E-07**
**rcc01648**	**ABC transporter permease**	**P**	**-2.73**	**5.87E-08**
rcc02536	cysT; sulfate ABC transporter permease	P	-2.57	3.71E-08
**rcc01650**	**ABC transporter permease**	**P**	**-2.56**	**7.21E-11**
rcc01723	ccpA; cytochrome-c peroxidase	P	-2.21	2.59E-16
**rcc02855**	**ABC transporter permease**	**P**	**-2.12**	**5.86E-05**
**rcc01727**	**pyrD1; dihydroorotate oxidase**	**P**	**2.07**	**2.20E-22**
rcc01033	cbiQ1; cobalt ABC transporter permease	P	2.08	1.65E-16
rcc01433	TonB-dependent siderophore receptor; K02014 iron comp	P	2.08	8.89E-08
rcc01434	fepB1; ferric enterobactin-binding periplasmic protein	P	2.20	5.35E-08
rcc00101	ABC transporter permease	P	2.23	4.11E-15
rcc01435	fepD1; ferric enterobactin transport system permease	P	2.31	2.86E-07
**rcc00021**	**cytochrome c peroxidase**	**P**	**2.35**	**1.12E-37**
rcc01049	TonB-dependent siderophore receptor	P	2.38	2.24E-09
rcc01436	fepG1; ferric enterobactin transport system permease	P	2.39	3.54E-05
**rcc02659**	**monosacharide ABC transporter ATP-binding protein**	**P**	**2.41**	**1.24E-35**
rcc00099	ABC transporter ATP-binding protein	P	2.42	7.34E-17
rcc00103	ABC transporter periplasmic substrate-binding protein	P	2.54	3.20E-23
rcc00102	ABC transporter permease	P	2.57	5.65E-25
rcc00100	ABC transporter ATP-binding protein	P	2.59	4.29E-27
rcc00150	hypothetical protein; K07227 hypothetical protein	P	3.25	3.06E-20
**rcc02311**	**sodB; superoxide dismutase**	**P**	**3.25**	**7.47E-77**
rcc00105	fhuC1; ferrichrome ABC transporter ATP-binding protein	P	3.41	1.37E-09
rcc00111	fhuE; outer membrane ferric siderophore receptor	P	3.49	9.04E-17
**rcc01028**	**iron siderophore/cobalamin ABC transporter**	**P**	**3.60**	**2.20E-12**
rcc00106	fhuB1; ferrichrome ABC transporter permease	P	3.68	1.50E-14
rcc01441	fepC2; ferric enterobactin transport ATP-binding protein	P	4.23	3.67E-25
**rcc03066**	**dyp-type peroxidase**	**P**	**4.78**	**3.60E-61**
rcc01445	tonB-dependent receptor	P	4.83	2.11E-19
**rcc03065**	**efeU; ferrous iron permease**	**P**	**4.85**	**1.61E-55**
rcc01046	iron siderophore/cobalamin ABC transporter permease	P	5.10	1.31E-23
rcc00107	fhuB2; ferrichrome ABC transporter permease	P	5.36	2.22E-31
rcc00108	fhuD1; ferrichrome ABC transporter	P	5.44	1.71E-28
rcc01442	fepG2; ferric enterobactin transport system permease	P	5.64	9.27E-24
rcc02579	Fe(III) ABC transporter permease	P	5.68	1.72E-81
rcc01443	fepD2; ferric enterobactin transport system permease	P	5.88	1.32E-19
rcc02473	TonB-dependent receptor plug domain-containing proteir	P	6.97	2.80E-102
rcc00094	hmuV; hemin ABC transporter ATP-binding protein	P	7.05	4.18E-41
rcc01444	fepB2; ferric enterobactin-binding periplasmic protein	P	7.11	6.32E-53
**rcc03067**	**EfeO; iron uptake system component**	**P**	**8.56**	**9.36E-178**

rcc02578	iron(III) ABC transporter periplasmic	P	13.77	1.44E-213
rcc00096	hmuT; hemin ABC transporter periplasmic hemin-binding	P	15.33	2.78E-67
rcc00095	hmuU; hemin ABC transporter permease	P	17.67	5.78E-96
rcc01047	iron siderophore/cobalamin ABC transporter	P	18.74	2.37E-171
rcc00098	hmuR; TonB-dependent hemin receptor	P	22.60	2.66E-178
rcc00097	hmuS; hemin transport protein HmuS	P	26.53	1.22E-165

COG Q: Secondary metabolites biosythesis, transport and catabolism

**rcc02016**	**fumarylacetoacetate hydrolase**	**Q**	**-7.45**	**1.68E-182**
rcc01546	AMP-dependent synthetase and ligase	Q	2.21	4.46E-38
rcc02110	multicopper oxidase	Q	2.38	0.001173778

COG R/S: General function prediction only/function unknown

**rcc02390**	**alkane 1-monooxygenase**	**S**	**-39.13**	**2.13E-221**
**rcc01232**	**hypothetical protein**	**S**	**-38.50**	**4.90E-53**
**rcc01233**	**hypothetical protein**	**S**	**-12.56**	**6.31E-72**
**rcc02463**	**hemolysin-type calcium-binding repeat family protein**	**R**	**-5.95**	**1.26E-77**
**rcc00542**	**hypothetical protein**	**S**	**-5.63**	**3.43E-71**
rcc00610	hypothetical protein	S	-5.23	1.12E-13
rcc02891	hypothetical protein	S	-4.87	1.77E-07
**rcc02815**	**lipoprotein**	**S**	**-4.81**	**3.53E-82**
**rcc03377**	**hypothetical protein**	**S**	**-4.70**	**3.08E-65**
**rcc01645**	**lipoprotein**	**R**	**-4.09**	**7.48E-13**
rcc02890	hypothetical protein	S	-3.85	1.83E-05
rcc00609	hypothetical protein	S	-3.77	9.04E-06
**rcc01654**	**hypothetical protein**	**S**	**-3.74**	**9.06E-20**
**rcc01653**	**YVTN beta-propeller repeat family protein**	**S**	**-3.72**	**6.87E-26**
**rcc01652**	**hypothetical protein**	**S**	**-3.64**	**4.36E-11**
rcc02005	hypothetical protein	S	-3.37	3.35E-67
**rcc00383**	**FAD dependent oxidoreductase**	**R**	**-3.27**	**1.88E-46**
**rcc01655**	**hypothetical protein**	**S**	**-3.25**	**2.31E-06**
rcc00530	hypothetical protein	S	-2.99	4.23E-72
**rcc03341**	**hypothetical protein**	**S**	**-2.99**	**2.78E-52**
**rcc03340**	**hypothetical protein**	**S**	**-2.94**	**1.33E-14**
**rcc03339**	**hypothetical protein**	**S**	**-2.88**	**2.59E-07**
rcc01273	hypothetical protein	S	-2.85	2.48E-27
rcc01410	hypothetical protein	S	-2.84	5.88E-18
rcc03008	hypothetical protein	S	-2.68	1.24E-30
rcc02892	hypothetical protein	S	-2.60	0.0009887
rcc03467	hypothetical protein	S	-2.57	3.73E-06
rcc03009	hypothetical protein	S	-2.53	5.85E-31
rcc00543	hypothetical protein	S	-2.48	6.91E-32
**rcc01408**	**hypothetical protein**	**S**	**-2.47**	**1.60E-05**
rcc02228	hypothetical protein	S	-2.43	0.001200653
**rcc01406**	**phage lysozyme**	**R**	**-2.36**	**7.28E-18**

**rcc01409**	**hypothetical protein**	**S**	**-2.29**	**1.23E-05**
rcc00435	hypothetical protein	S	-2.28	8.48E-14
rcc02014	hypothetical protein	S	-2.16	7.33E-16
rcc00842	hypothetical protein	S	-2.15	2.20E-16
rcc02385	hypothetical protein	S	-2.13	2.96E-14
rcc00655	hypothetical protein	S	-2.13	1.61E-21
rcc02189	hypothetical protein	S	-2.09	2.13E-14
rcc00178	hemolysin-type calcium-binding repeat family protein	S	2.01	1.42E-12
rcc00048	metal dependent phosphohydrolase	R	2.02	4.63E-24
rcc02187	amidohydrolase	R	2.03	1.64E-12
rcc01044	RimK-like ATP-grasp domain-containing protein	S	2.04	1.01E-09
**rcc03403**	**hypothetical protein**	**S**	**2.04**	**2.85E-12**
**rcc00642**	**peptidoglycan binding domain-containing protein**	**S**	**2.06**	**4.14E-18**
rcc00910	hypothetical protein	S	2.09	4.54E-17
rcc03124	hypothetical protein	S	2.17	5.79E-24
rcc02109	hypothetical protein	S	2.24	6.76E-10
rcc01234	hypothetical protein	S	2.26	1.00E-13
**rcc02658**	**lipoprotein**	**S**	**2.32**	**8.22E-28**
**rcc01721**	**TfoX domain-containing protein**	**S**	**2.43**	**1.81E-14**
**rcc00423**	**hypothetical protein**	**S**	**2.46**	**3.22E-19**
rcc00109	hypothetical protein	S	2.51	5.37E-09
rcc01038	hypothetical protein	S	2.52	4.68E-34
**rcc00885**	**hypothetical protein**	**S**	**2.55**	**8.00E-08**
**rcc02646**	**hypothetical protein**	**S**	**2.57**	**2.15E-40**
**rcc00891**	**hypothetical protein**	**S**	**2.58**	**4.66E-18**
**rcc02141**	**hypothetical protein**	**S**	**2.69**	**1.28E-13**
**rcc00104**	**hypothetical protein**	**S**	**2.70**	**2.00E-25**
**rcc02293**	**hypothetical protein**	**S**	**2.72**	**4.22E-11**
rcc01036	hypothetical protein	S	2.76	2.67E-07
**rcc01423**	**hypothetical protein**	**S**	**2.77**	**1.43E-39**
**rcc00890**	**hypothetical protein**	**S**	**2.78**	**1.48E-12**
**rcp00068**	**M4 family peptidase**	**S**	**2.92**	**5.90E-16**
**rcc01035**	**hypothetical protein**	**S**	**3.06**	**5.42E-44**
**rcp00077**	**hypothetical protein**	**S**	**3.18**	**5.61E-17**
rcc01037	hypothetical protein	S	3.25	2.40E-18
rcc00110	esterase; K07017	R	3.27	3.96E-17
rcc00087	hypothetical protein	S	3.53	4.21E-28
**rcc02119**	**type 12 family methyltransferase**	**R**	**3.70**	**4.70E-56**
**rcc01027**	**hypothetical protein**	**S**	**4.09**	**2.04E-18**
rcc01043	hypothetical protein	S	4.33	5.09E-57
**rcc02118**	**cat; chloramphenicol acetyltransferase**	**R**	**4.35**	**4.47E-76**
rcc02474	hypothetical protein	S	4.51	9.05E-49
**rcc02454**	**PAS domain-containing protein**	**S**	**4.92**	**1.54E-18**
**rcc03429**	**hypothetical protein**	**S**	**5.87**	**3.43E-140**
**rcp00069**	**hypothetical protein**	**S**	**7.35**	**2.12E-76**

rcc03162	mandelate racemase/muconate lactonizing enzyme	S	9.58	1.30E-279
rcc01112	hypothetical protein	S	12.20	1.59E-44
rcc01111	hypothetical protein	S	12.57	1.63E-48
rcc03163	hypothetical protein	S	36.35	0

COG T: Signal transduction mechanisms

**rcc00045**	**regA1; photosynthetic apparatus regulatory protein**	**T**	**-69.16**	**0**
**rcc00567**	**nifA1; Nif-specific regulatory protein**	**T**	**-5.76**	**6.31E-28**
**rcc01020**	**diguanylate cyclase/phosphodiesterase**	**T**	**-3.04**	**5.45E-27**
rcc03031	PspA/IM30 family protein	T,K	-2.52	9.28E-14
rcc00681	tspO; signal transduction protein	T	-2.51	1.79E-32
**rcc00356**	**cyclic nucleotide-binding domain-containing protein**	**T**	**-2.49**	**9.74E-32**
**rcc02856**	**PAS/PAC sensor domain-containing protein**	**T**	**-2.46**	**1.90E-30**
**rcc02857**	**diguanylate cyclase/phosphodiesterase**	**T**	**-2.04**	**1.40E-31**
rcc00645	diguanylate cyclase/phosphodiesterase	T	2.50	1.85E-46
**rcc01495**	**fusA2; translation elongation factor G**	**T**	**2.69**	**2.25E-24**
**rcc00643**	**diguanylate cyclase/phosphodiesterase**	**T**	**2.71**	**2.26E-51**
**rcc02294**	**signal transduction histidine kinase**	**T**	**3.00**	**2.07E-48**
**rcc01156**	**UspA domain-containing protein**	**T**	**3.70**	**1.59E-09**

COG U: Intracellular trafficking, secretion, and vesicular transport

rcc02375	exbB; biopolymer transport protein ExbB	U	2.53	1.52E-26
rcc02376	exbD1; biopolymer transport protein ExbD	U	2.58	1.55E-12

COG V: Defense mechanisms

**rcc00886**	**ABC transporter ATP-binding protein**	**V**	**2.93**	**2.01E-07**
rcc01439	ABC transporter ATP-binding/permease	V	3.50	6.79E-40
rcc01440	ABC transporter ATP-binding/permease	V	8.16	1.33E-73

Table S2: WT-Δ*regA* DEGs under photosynthetic conditions in RCV organized by COG and regulation strength. Genes highlighted in red indicate repression by RegA, genes highlighted in green indicate activation by RegA. Genes highlighted in bold-face type indicate that they are regulated in both PY and RCV medium.

Table S3: >4fold WT-Δ*regA* DEGs under photosynthetic conditions

		RCV		PY	
Gene		FC	p-val	FC	p-val
COG C: Energy production and storage					
rcc01646	cytochrome c domain-containing protein	-4.11	1.19E-22	-14.02	5.46E-07
rcc02015	aldH1; aldehyde dehydrogenase	-6.88	3.31E-142	-10.96	1.28E-139
rcc02531	pucA; light-harvesting protein B-800/850 subunit alpha	-3.59	1.83E-65	-5.63	3.96E-79
rcc02530	pucB; light-harvesting protein B-800/850 subunit beta	-5.34	5.33E-122	-5.32	3.35E-79
rcp00070	nosX; NosX protein	9.28	3.71E-72	NA	NA
rcp00071	nosL; nitrous oxide reductase accessory protein	13.73	9.72E-78	NA	NA
rcp00072	nosY; cooper ABC transporter permease	6.33	7.98E-47	3.32	5.99E-06
rcp00074	nosD; nitrous oxide maturation protein NosD	9.90	3.43E-114	4.01	0.00102436
rcp00073	nosF; copper ABC transporter ATP-binding protein NosF	10.02	6.88E-46	4.23	0.00053782
rcp00075	nosZ; nitrous-oxide reductase	13.15	3.10E-216	3.81	0.00964983
rcc02866	pflB; formate C-acetyltransferase	NA	NA	4.27	1.61E-11
rcc01161	ccoG; cbb3-type cytochrome c oxidase accessory protein	3.34	2.89E-81	4.55	5.25E-35
rcc02248	aldo/keto reductase family oxidoreductase	NA	NA	5.23	4.67E-19
rcc01729	pyridine nucleotide-disulfide oxidoreductase	NA	NA	5.32	1.63E-16
rcc00767	hupA; hydrogenase small subunit	7.64	2.33E-171	7.76	1.74E-33
rcc01728	nifJ; pyruvate-flavodoxin oxidoreductase	3.67	1.60E-70	8.76	1.40E-24
rcc00768	hupB; hydrogenase large subunit	7.94	7.49E-168	9.67	6.86E-56
rcc00769	hupC; hydrogenase, cytochrome b subunit	7.79	3.39E-101	11.05	6.49E-67
rcc01160	ccoP; cbb3-type cytochrome c oxidase subunit III	9.00	1.92E-250	11.37	5.79E-46
rcc01157	ccoN; cbb3-type cytochrome c oxidase subunit I	8.39	2.23E-232	12.48	6.23E-73
rcc01158	ccoO; cbb3-type cytochrome c oxidase subunit II	9.66	4.48E-239	12.75	3.96E-59
rcc01159	ccoQ; cbb3-type cytochrome c oxidase subunit IV	10.07	2.34E-230	13.08	8.00E-62
rcc02847	torC; trimethylamine-N-oxide reductase c-type cytochrome	NA	NA	31.65	7.88E-84
rcc02845	torA; trimethylamine-N-oxide reductase	NA	NA	33.85	5.68E-103
rcc02846	torD; chaperone protein TorD	NA	NA	34.07	9.59E-23
COG E: Amino acid transport and metabolism					
rcc01072	hutH; histidine ammonia-lyase	NA	NA	-8.98	4.39E-50
rcc00516	tpl; tyrosine phenol-lyase	-9.65	1.01E-173	-8.92	2.52E-49
rcc03430	livM3; branched-chain amino acid ABC transporter permease	NA	NA	4.59	2.45E-15
rcc02274	pip; proline iminopeptidase	NA	NA	6.80	1.72E-19
rcc02275	oppA2; oligopeptide ABC transporter periplasmic protein	NA	NA	7.15	5.80E-53
rcc02452	potD4; polyamine ABC transporter periplasmic protein	4.28	1.81E-75	3.55	7.67E-13
rcc02451	potA4; polyamine ABC transporter ATP-binding protein	4.53	2.75E-22	2.11	0.00728307
rcc00484	ald; alanine dehydrogenase	60.53	0	3.70	3.41E-26
COG G: Carbohydrate transport and metabolism					
rcc03013	DeoC/LacD family aldolase	-4.77	2.86E-55	-4.48	4.79E-32
rcc01657	exaA2; quinoprotein ethanol dehydrogenase	-2.88	3.25E-41	-4.03	1.97E-07
rcc02654	tktB; transketolase, C-terminal subunit	NA	NA	4.08	7.30E-38
rcc02660	monosacharide ABC transporter permease	2.46	1.01E-31	4.77	9.61E-60
rcc02657	monosacharide ABC transporter periplasmic protein	2.58	2.67E-58	6.13	3.51E-89
COG GRP: Glycyl radical enzyme microcompartment					
rcc02200	hypothetical protein	NA	NA	76.33	3.10E-55
rcc02202	hypothetical protein	NA	NA	84.38	8.95E-28
rcc02199	pflA1; [pyruvate formate-lyase]-activating enzyme	NA	NA	88.70	5.14E-100
rcc02214	ackA2; acetate kinase	NA	NA	96.18	3.60E-133
rcc02210	adh2; aldehyde-alcohol dehydrogenase 2	NA	NA	97.00	4.55E-100

rcc02201	adhE; aldehyde-alcohol dehydrogenase	NA	NA	103.54	1.06E-148
rcc02204	hypothetical protein	NA	NA	110.00	1.10E-32
rcc02203	eutN; ethanolamine utilization protein EutN	NA	NA	110.29	1.05E-42
rcc02207	pduA1; propanediol utilization protein PduA	NA	NA	110.84	1.62E-72
rcc02209	pduA2; propanediol utilization protein PduA	NA	NA	112.32	2.12E-37
rcc02205	eutJ; ethanolamine utilization protein EutJ	NA	NA	129.92	2.72E-71
rcc02206	pduL; propanediol utilization protein PduL	NA	NA	141.22	7.07E-109
rcc02208	pduB; propanediol utilization protein PduB	2.01	0.00690814	196.48	5.05E-141
rcc02213	hypothetical protein	NA	NA	207.04	2.42E-149
rcc02212	ThiJ/PfpI family protein; K05520 protease I	NA	NA	248.49	2.41E-191
rcc02211	pflD; formate C-acetyltransferase	NA	NA	251.31	0

COG H: Coenzyme transport and metabolism

rcc00028	idi1; isopentenyl-diphosphate delta-isomerase	-6.09	1.78E-41	-8.33	3.84E-24
rcp00134	citG; triphosphoribosyl-dephospho-CoA synthase	-2.00	0.00519485	-4.46	0.0141034
rcc01235	pyrimidine 5'-nucleotidase	-2.48	1.74E-06	-4.27	4.84E-15
rcc02840	moaC2; molybdenum cofactor biosynthesis protein C	NA	NA	8.33	1.06E-22
rcc02843	moaA2; molybdenum cofactor biosynthesis protein A	NA	NA	14.47	2.45E-53
rcc02841	moeB2; molybdenum cofactor biosynthesis protein B	NA	NA	16.27	2.20E-53
rcc02842	moaD1; molybdenum cofactor biosynthesis protein D	NA	NA	18.00	2.99E-22
rcc00151	hemN1; oxygen-independent coproporphyrinogen-III oxidase	4.44	1.77E-34	NA	NA

COG I: Lipid transport and metabolism

rcc01063	pcl; 4-coumarate--CoA ligase	NA	NA	-4.62	1.78E-09

COG K: Transcription

rcc02675	MarR family transcriptional regulator	NA	NA	-7.78	4.94E-88
rcc01722	BadM/Rrf2 family transcriptional regulator	-4.25	8.96E-44	-3.66	3.47E-10
rcp00076	nosR; nitrous-oxide reductase expression regulator NosR	5.78	8.05E-78	5.03	3.10E-20
rcc02453	Fis family sigma54 specific transcriptional regulator	6.04	8.30E-42	5.13	6.84E-11
rcc00112	AraC family transcriptional regulator	4.19	2.57E-15	NA	NA
rcc01048	AraC family transcriptional regulator	4.88	2.09E-16	NA	NA

COG L: Replication, recombination, and repair

rcc02864	DNA polymerase III subunit epsilon	NA	NA	8.69	1.38E-08

COG M: Cell wall/membrane/envelope biogenesis

rcc00055	transglycosylase, Slt family	NA	NA	-21.57	7.75E-71
rcc01697	NlpC/P60 family phage cell wall peptidase	NA	NA	-4.70	0.00368448
rcc00887	hypothetical protein	2.89	1.18E-13	6.03	4.02E-26

COG N: Cell motility

rcc00056	flhA; flagellar biosynthesis protein FlhA	NA	NA	-29.15	5.62E-163
rcc03523	flbT; flagellin synthesis repressor protein FlbT	NA	NA	-22.91	3.49E-176
rcc01767	cheX; chemotaxis protein CheX	NA	NA	-21.75	1.34E-139
rcc03014	methyl-accepting chemotaxis sensory transducer	NA	NA	-20.44	7.73E-189
rcc00057	fliR; flagellar biosynthetic protein FliR	NA	NA	-20.17	2.82E-106
rcc00759	mcpB; methyl-accepting chemotaxis protein McpB	NA	NA	-20.15	4.70E-165
rcc01766	cheY3; chemotaxis protein CheY	NA	NA	-19.95	3.90E-176
rcc03524	flagellar FlaF family protein	NA	NA	-18.48	6.98E-134
rcc03522	flagellar protein	NA	NA	-16.38	3.50E-114
rcc00058	flhB; flagellar biosynthetic protein FlhB	NA	NA	-15.81	1.73E-97
rcc01765	cheA2; chemotaxis protein CheA	NA	NA	-15.50	2.92E-161

**rcc01763**	**cheR3; chemotaxis protein methyltransferase CheR**	NA	NA	**-15.47**	**2.10E-132**
**rcc01764**	**cheW2; chemotaxis protein CheW**	NA	NA	**-14.44**	**1.69E-132**
**rcc03483**	**fliL1; flagellar basal body-associated protein FliL**	NA	NA	**-14.34**	**4.19E-117**
**rcc00760**	**mcpA1; methyl-accepting chemotaxis protein McpA**	NA	NA	**-14.20**	**1.40E-102**
**rcc02151**	**methyl-accepting chemotaxis sensory transducer**	**-4.25**	**3.21E-39**	**-13.21**	**9.27E-71**
**rcc01759**	**cheB2; chemotaxis response regulator protein**	NA	NA	**-13.13**	**3.16E-86**
**rcc01762**	**cheY2; chemotaxis protein CheY**	NA	NA	**-13.06**	**3.55E-100**
**rcc03515**	**flgG; flagellar basal-body rod protein FlgG**	NA	NA	**-12.51**	**2.33E-117**
**rcc03516**	**flgF; flagellar basal-body rod protein FlgF**	NA	NA	**-12.10**	**1.85E-128**
**rcc03514**	**flgA; flagella basal body P-ring formation protein FlgA**	NA	NA	**-11.50**	**2.89E-89**
**rcc00007**	**flgE; flagellar hook protein FlgE**	NA	NA	**-11.48**	**2.56E-130**
**rcc03481**	**fliH; flagellar biosynthesis/type III secretory pathway protein**	NA	NA	**-11.28**	**3.03E-75**
**rcc03513**	**flgH; flagellar L-ring protein FlgH**	NA	NA	**-10.94**	**7.15E-118**
**rcc03486**	**motA; chemotaxis protein MotA**	NA	NA	**-10.85**	**2.93E-146**
**rcc03517**	**fliQ; flagellar biosynthetic protein FliQ**	NA	NA	**-10.84**	**6.02E-103**
**rcc03520**	**flgB; flagellar basal-body rod protein; FlgB**	NA	NA	**-10.79**	**3.00E-118**
**rcc03525**	**flaA; flagellin protein**	NA	NA	**-10.51**	**7.91E-126**
**rcc02611**	**mcpA3; methyl-accepting chemotaxis protein McpA**	NA	NA	**-10.12**	**2.23E-100**
**rcc03519**	**flgC; flagellar basal-body rod protein FlgC**	NA	NA	**-10.09**	**1.20E-107**
**rcc01073**	**gvpA; gas vesicle protein GvpA**	NA	NA	**-10.08**	**1.76E-40**
**rcc03482**	**fliF; flagellar M-ring protein FliF**	NA	NA	**-10.05**	**7.28E-166**
**rcc03518**	**fliE; flagellar hook-basal body complex protein FliE**	NA	NA	**-9.40**	**3.57E-102**
**rcc01760**	**cheD; chemoreceptor glutamine deamidase CheD**	NA	NA	**-9.32**	**9.87E-47**
**rcc01758**	**mcpA2; methyl-accepting chemotaxis protein McpA**	NA	NA	**-9.19**	**3.20E-102**
**rcc02887**	**methyl-accepting chemotaxis sensory transducer**	NA	NA	**-8.33**	**2.18E-73**
**rcc01354**	**cheW1; chemotaxis protein CheW**	NA	NA	**-8.19**	**9.25E-39**
**rcc01053**	**gvpO; gas vesicle protein GvpO**	NA	NA	**-8.18**	**3.20E-11**
**rcc01355**	**methyl-accepting chemotaxis sensory transducer**	NA	NA	**-7.86**	**4.60E-73**
**rcc01051**	**gvpN; gas vesicle protein GvpN**	NA	NA	**-7.76**	**6.55E-31**
**rcc00009**	**flgL; flagellar hook-associated protein FlgL**	NA	NA	**-7.73**	**7.72E-89**
**rcc03480**	**fliN; flagellar motor switch protein FliN**	NA	NA	**-7.45**	**1.67E-42**
**rcc01353**	**cheA1; chemotaxis protein CheA**	NA	NA	**-7.28**	**1.55E-40**
**rcc00008**	**flgK; flagellar hook-associated protein FlgK**	NA	NA	**-7.26**	**1.82E-76**
**rcc01357**	**cheR2; chemotaxis protein methyltransferase CheR**	NA	NA	**-7.20**	**2.22E-51**
**rcc01054**	**gvpJ; gas vesicle protein GvpJ**	NA	NA	**-6.97**	**5.55E-13**
**rcc01352**	**cheY1; chemotaxis protein CheY**	NA	NA	**-6.92**	**5.04E-34**
**rcc01356**	**chemotaxis protein CheW**	NA	NA	**-6.86**	**4.25E-39**
**rcc00010**	**flgI; flagellar P-ring protein FlgI**	NA	NA	**-6.80**	**4.30E-106**
**rcc03521**	**fliI; flagellar protein export ATPase FliI**	NA	NA	**-6.73**	**2.49E-58**
**rcc01056**	**gas vesicle synthesis protein GvpL/GvpF**	NA	NA	**-6.45**	**3.82E-19**
**rcc03512**	**fliL2; flagellar basal body-associated protein FliL**	NA	NA	**-6.23**	**1.56E-66**
**rcc02158**	**fliG; flagellar motor switch protein FliG**	NA	NA	**-6.16**	**6.93E-58**
**rcc01075**	**methyl-accepting chemotaxis protein**	NA	NA	**-6.09**	**2.41E-26**
**rcc03529**	**flgD; flagellar hook capping protein**	NA	NA	**-5.47**	**2.70E-59**
**rcc01358**	**cheB1; chemotaxis response regulator**	NA	NA	**-5.28**	**3.79E-26**
**rcc01057**	**gvpG; gas vesicle protein GvpG**	NA	NA	**-5.25**	**4.65E-12**
**rcc03527**	**flgJ; flagellar protein FlgJ**	NA	NA	**-5.09**	**1.26E-39**
**rcc00644**	**mcpX; methyl-accepting chemotaxis protein McpX**	NA	NA	**-4.87**	**1.14E-23**

COG O: Posttranslational modification, protein turnover, chaperones

**rcc02069**	**hypothetical protein**	NA	NA	**-15.08**	**3.84E-104**
**rcc00771**	**hupF; hydrogenase maturation chaperone HupF**	2.90	1.53E-20	**4.61**	**1.61E-09**
**rcc00775**	**hupK; hydrogenase expression/formation protein HupK**	2.73	1.86E-22	**4.77**	**1.29E-19**

**rcc00773**	**hupH; hydrogenase expression/formation protein HupH**	2.70	3.50E-39	**4.77**	**2.85E-50**
**rcc02865**	**pflA2; [pyruvate formate-lyase]-activating enzyme**	NA	NA	**5.18**	**1.06E-13**

COG P: Inorganic ion transport and metabolism

**rcc01647**	**ABC transporter periplasmic substrate-binding protein**	-3.81	5.95E-18	**-9.05**	**2.63E-10**
**rcc03358**	**TonB-dependent receptor**	-3.15	5.57E-07	**-7.01**	**6.76E-31**
**rcc03359**	**iron siderophore/cobalamin ABC transporter**	-3.24	2.87E-07	**-6.28**	**1.00E-15**
**rcc02659**	**monosacharide ABC transporter ATP-binding protein**	2.41	1.24E-35	**4.78**	**5.11E-63**
**rcc01028**	**iron siderophore/cobalamin ABC transporter protein**	3.60	2.20E-12	**5.75**	**1.77E-27**
**rcc01441**	**fepC2; ferric enterobactin transport ATP-binding protein**	**4.23**	**3.67E-25**	NA	NA
**rcc03066**	**dyp-type peroxidase**	**4.78**	**3.60E-61**	2.80	4.89E-15
**rcc01445**	**tonB-dependent receptor**	**4.83**	**2.11E-19**	NA	NA
**rcc03065**	**efeU; ferrous iron permease**	**4.85**	**1.61E-55**	2.27	8.98E-06
**rcc01046**	**iron siderophore/cobalamin ABC transporter permease**	**5.10**	**1.31E-23**	NA	NA
**rcc00107**	**fhuB2; ferrichrome ABC transporter permease**	**5.36**	**2.22E-31**	NA	NA
**rcc00108**	**fhuD1; ferrichrome ABC transporter**	**5.44**	**1.71E-28**	NA	NA
**rcc01442**	**fepG2; ferric enterobactin transport system permease**	**5.64**	**9.27E-24**	NA	NA
**rcc02579**	**Fe(III) ABC transporter permease**	**5.68**	**1.72E-81**	NA	NA
**rcc01443**	**fepD2; ferric enterobactin transport system permease**	**5.88**	**1.32E-19**	NA	NA
**rcc02473**	**TonB-dependent receptor plug domain-containing protein**	**6.97**	**2.80E-102**	NA	NA
**rcc00094**	**hmuV; hemin ABC transporter ATP-binding protein**	**7.05**	**4.18E-41**	NA	NA
**rcc01444**	**fepB2; ferric enterobactin-binding periplasmic protein**	**7.11**	**6.32E-53**	NA	NA
**rcc03067**	**EfeO; iron uptake system component**	**8.56**	**9.36E-178**	2.66	4.17E-14
**rcc02578**	**iron(III) ABC transporter periplasmic**	**13.77**	**1.44E-213**	NA	NA
**rcc00096**	**hmuT; hemin ABC transporter periplasmic protein**	**15.33**	**2.78E-67**	NA	NA
**rcc00095**	**hmuU; hemin ABC transporter permease**	**17.67**	**5.78E-96**	NA	NA
**rcc01047**	**iron siderophore/cobalamin ABC transporter**	**18.74**	**2.37E-171**	NA	NA
**rcc00098**	**hmuR; TonB-dependent hemin receptor**	**22.60**	**2.66E-178**	NA	NA
**rcc00097**	**hmuS; hemin transport protein HmuS**	**26.53**	**1.22E-165**	NA	NA

COG Q: Secondary metabolites biosynthesis, transport and catabolism

**rcc02016**	**fumarylacetoacetate hydrolase**	**-7.45**	**1.68E-182**	**-14.30**	**4.54E-131**

COG R/S: General function prediction only/function unknown

**rcc01691**	**TP901-1 family phage major tail protein**	NA	NA	**-12.59**	**1.68E-14**
**rcc01684**	**HK97 family phage portal protein**	NA	NA	**-12.47**	**5.45E-16**
**rcc01687**	**HK97 family phage major capsid protein**	NA	NA	**-11.59**	**1.44E-11**
**rcc01686**	**phage prohead protease**	NA	NA	**-8.90**	**1.92E-08**
**rcc00197**	**comF; competence protein F**	NA	NA	**-8.07**	**3.99E-14**
**rcc01645**	**lipoprotein**	**-4.09**	**7.48E-13**	**-4.78**	**2.68E-06**
**rcc01232**	**hypothetical protein**	**-38.50**	**4.90E-53**	**-68.61**	**6.61E-61**
**rcc02390**	**alkane 1-monooxygenase**	**-39.13**	**2.13E-221**	**-37.10**	**1.02E-95**
**rcc01688**	**hypothetical protein**	NA	NA	**-22.36**	**1.77E-19**
**rcc02067**	**type 11 family methyltransferase**	NA	NA	**-19.82**	**3.08E-116**
**rcc01695**	**hypothetical protein**	NA	NA	**-14.65**	**8.73E-15**
**rcc02068**	**ice nucleation protein repeat family protein**	NA	NA	**-13.06**	**4.01E-100**
**rcc02063**	**M10 family peptidase**	NA	NA	**-12.58**	**1.88E-67**
**rcc00630**	**ice nucleation protein repeat family protein**	NA	NA	**-12.22**	**1.68E-81**
**rcc01761**	**hypothetical protein**	NA	NA	**-11.31**	**1.83E-104**
**rcc02591**	**surface presentation of antigens protein family**	NA	NA	**-11.08**	**9.51E-80**
**rcc03485**	**hypothetical protein**	NA	NA	**-10.78**	**1.39E-117**
**rcc02610**	**hypothetical protein**	NA	NA	**-10.38**	**3.65E-58**
**rcc01690**	**hypothetical protein**	NA	NA	**-10.17**	**2.68E-07**

**rcc03487**	**hypothetical protein**	NA	NA	**-10.05**	**3.77E-115**
**rcc01076**	**hypothetical protein**	NA	NA	**-9.85**	**5.15E-33**
**rcc01350**	**hypothetical protein**	NA	NA	**-9.34**	**5.99E-58**
**rcc03528**	**hypothetical protein**	NA	NA	**-9.23**	**1.22E-117**
**rcc01696**	**hypothetical protein**	NA	NA	**-8.67**	**4.93E-11**
**rcc03377**	**hypothetical protein**	**-4.70**	**3.08E-65**	**-8.55**	**1.02E-62**
**rcc02623**	**hypothetical protein**	NA	NA	**-8.21**	**5.06E-09**
**rcc01694**	**hypothetical protein**	NA	NA	**-8.06**	**0.00029779**
**rcc01865**	**hypothetical protein**	NA	NA	**-7.78**	**3.21E-14**
**rcc01652**	**hypothetical protein**	-3.64	4.36E-11	**-7.75**	**3.66E-09**
**rcc03484**	**hypothetical protein**	NA	NA	**-7.65**	**9.83E-38**
**rcc01685**	**hypothetical protein**	NA	NA	**-7.54**	**0.0001143**
**rcc01138**	**hypothetical protein**	NA	NA	**-7.30**	**5.96E-28**
**rcc01918**	**hypothetical protein**	NA	NA	**-7.29**	**0.00266872**
**rcc01080**	**hypothetical protein**	NA	NA	**-6.89**	**2.06E-12**
**rcc03299**	**hypothetical protein**	NA	NA	**-6.83**	**2.60E-23**
**rcc01233**	**hypothetical protein**	**-12.56**	**6.31E-72**	**-6.54**	**3.65E-05**
**rcc02415**	**hypothetical protein**	NA	NA	**-6.51**	**2.10E-39**
**rcc02488**	**hypothetical protein**	NA	NA	**-6.22**	**5.61E-35**
**rcc01139**	**hypothetical protein**	NA	NA	**-6.14**	**1.58E-35**
**rcc03526**	**hypothetical protein**	NA	NA	**-6.13**	**3.47E-71**
**rcp00010**	**hypothetical protein**	NA	NA	**-6.01**	**0.01383397**
**rcc01067**	**hypothetical protein**	NA	NA	**-5.84**	**1.76E-40**
**rcc01698**	**hypothetical protein**	NA	NA	**-5.80**	**4.50E-16**
**rcc01917**	**hypothetical protein**	NA	NA	**-5.79**	**0.0003599**
**rcc01074**	**hypothetical protein**	NA	NA	**-5.65**	**0.00245479**
**rcc03122**	**FRG domain-containing protein**	NA	NA	**-5.60**	**3.19E-32**
**rcc00142**	**hypothetical protein**	NA	NA	**-5.48**	**1.31E-63**
**rcc01079**	**hypothetical protein**	NA	NA	**-5.41**	**1.13E-09**
**rcc01070**	**hypothetical protein**	NA	NA	**-5.36**	**1.33E-07**
**rcc01066**	**pyp; photoactive yellow protein**	NA	NA	**-5.31**	**3.69E-34**
**rcc01242**	**hemolysin-type calcium-binding repeat family protein**	NA	NA	**-5.25**	**7.89E-25**
**rcc01064**	**hypothetical protein**	NA	NA	**-5.23**	**1.59E-27**
**rcc01065**	**hypothetical protein**	NA	NA	**-5.22**	**4.22E-13**
**rcc01653**	**YVTN beta-propeller repeat family protein**	-3.72	6.87E-26	**-5.13**	**7.27E-10**
**rcc01055**	**hypothetical protein**	NA	NA	**-5.12**	**2.98E-09**
**rcc02630**	**heme NO binding domain-containing protein**	NA	NA	**-4.94**	**1.11E-22**
**rcc02172**	**hypothetical protein**	NA	NA	**-4.82**	**1.74E-12**
**rcc02815**	**lipoprotein**	**-4.81**	**3.53E-82**	**-4.72**	**1.09E-21**
**rcc02980**	**hypothetical protein**	NA	NA	**-4.70**	**1.28E-27**
**rcc00844**	**hypothetical protein**	NA	NA	**-4.64**	**1.50E-27**
**rcc01655**	**hypothetical protein**	-3.25	2.31E-06	**-4.60**	**0.00021625**
**rcc03341**	**hypothetical protein**	-2.99	2.78E-52	**-4.47**	**4.84E-13**
**rcc03340**	**hypothetical protein**	-2.94	1.33E-14	**-4.44**	**6.21E-10**
**rcc01654**	**hypothetical protein**	-3.74	9.06E-20	**-4.40**	**4.78E-05**
**rcc00216**	**hypothetical protein**	NA	NA	**-4.20**	**1.77E-19**
**rcc01071**	**hypothetical protein**	NA	NA	**-4.13**	**6.90E-06**
**rcc01507**	**hypothetical protein**	NA	NA	**-4.03**	**7.37E-26**
**rcc01351**	**hypothetical protein**	NA	NA	**-4.03**	**5.85E-07**
**rcc00482**	**hypothetical protein**	NA	NA	**-4.00**	**6.78E-11**
**rcc02463**	**hemolysin-type calcium-binding repeat family protein**	**-5.95**	**1.26E-77**	-2.84	1.51E-11
**rcc00542**	**hypothetical protein**	**-5.63**	**3.43E-71**	-3.55	2.66E-14
**rcc00610**	**hypothetical protein**	**-5.23**	**1.12E-13**	NA	NA

rcc02891	hypothetical protein	-4.87	1.77E-07	NA	NA
rcc00965	phage tail assembly protein	NA	NA	4.01	6.59E-05
rcc02684	polyphosphate kinase 2 domain-containing protein	NA	NA	4.06	3.94E-13
rcc00989	lysozyme	NA	NA	4.08	1.39E-16
rcc00986	hypothetical protein	NA	NA	4.08	2.79E-14
rcc00987	hypothetical protein	NA	NA	4.11	5.02E-07
rcc00424	hypothetical protein	NA	NA	4.15	6.54E-14
rcc02658	lipoprotein	2.32	8.22E-28	4.41	3.30E-52
rcc00988	hypothetical protein	NA	NA	4.52	1.46E-10
rcc00978	hypothetical protein	NA	NA	4.70	4.30E-14
rcc00977	hypothetical protein	NA	NA	4.73	2.21E-23
rcc00973	hypothetical protein	NA	NA	4.77	1.30E-09
rcc00972	hypothetical protein	NA	NA	5.01	0.00055693
rcc02195	HNH endonuclease	NA	NA	5.06	1.94E-30
rcc00979	hypothetical protein	NA	NA	5.09	3.75E-31
rcc00976	hypothetical protein	NA	NA	5.14	3.57E-20
rcc03429	hypothetical protein	5.87	3.43E-140	5.20	2.26E-06
rcc02454	PAS domain-containing protein	4.92	1.54E-18	5.34	1.27E-09
rcc03403	hypothetical protein	2.04	2.85E-12	5.50	2.05E-16
rcc00888	hypothetical protein	NA	NA	5.55	3.95E-10
rcc00974	hypothetical protein	NA	NA	5.80	2.09E-06
rcc00970	hypothetical protein	NA	NA	5.90	7.01E-18
rcc00890	hypothetical protein	2.78	1.48E-12	5.94	7.28E-23
rcc00891	hypothetical protein	2.58	4.66E-18	5.99	1.07E-27
rcc00423	hypothetical protein	2.46	3.22E-19	6.28	4.96E-18
rcp00077	hypothetical protein	3.18	5.61E-17	6.49	0.00017152
rcc00889	NosL family protein	NA	NA	7.27	1.39E-35
rcc01897	hypothetical protein	NA	NA	7.30	1.38E-16
rcc00737	hypothetical protein	NA	NA	7.98	1.49E-36
rcc02141	hypothetical protein	2.69	1.28E-13	8.07	1.07E-23
rcp00069	hypothetical protein	7.35	2.12E-76	9.28	4.93E-16
rcp00067	hypothetical protein	NA	NA	10.39	2.30E-14
rcc01027	hypothetical protein	4.09	2.04E-18	11.07	1.48E-37
rcc02764	hypothetical protein	NA	NA	11.59	1.29E-69
rcc02273	hypothetical protein	NA	NA	13.97	1.49E-31
rcc02844	hypothetical protein	NA	NA	16.20	3.73E-22
rcc01043	hypothetical protein	4.33	5.09E-57	NA	NA
rcc02474	hypothetical protein	4.51	9.05E-49	NA	NA
rcc03162	mandelate racemase/muconate lactonizing enzyme	9.58	1.30E-279	NA	NA
rcc01112	hypothetical protein	12.20	1.59E-44	NA	NA
rcc01111	hypothetical protein	12.57	1.63E-48	NA	NA
rcc03163	hypothetical protein	36.35	0	NA	NA

COG T: Signal transduction mechanisms

rcc00045	regA1; photosynthetic apparatus regulatory protein RegA	-69.16	0	-73.77	1.15E-228
rcc02856	PAS/PAC sensor domain-containing protein	-2.46	1.90E-30	-34.31	2.25E-118
rcc03323	rsbV; anti-sigma-factor antagonist	NA	NA	-22.08	2.09E-89
rcc02857	diguanylate cyclase/phosphodiesterase	-2.04	1.40E-31	-12.81	1.40E-103
rcc00181	response regulator receiver domain	NA	NA	-12.65	1.77E-63
rcc03452	sensor histidine kinase/response regulator receiver protein	NA	NA	-11.80	8.22E-64
rcc00620	response regulator; diguanylate cyclase/phosphodiesterase	NA	NA	-11.25	1.44E-92
rcc00537	response regulator receiver protein	NA	NA	-10.16	1.93E-84
rcp00137	PAS/PAC sensor domain-containing protein	NA	NA	-9.88	1.84E-67

**rcc03177**	**EAL domain-containing protein**	NA	NA	**-9.02**	**4.52E-59**
**rcc02070**	**ArsR family transcriptional regulator**	NA	NA	**-8.25**	**0.00403614**
**rcc02539**	**diguanylate cyclase/phosphodiesterase**	NA	NA	**-7.68**	**4.53E-40**
**rcc00042**	**PAS/PAC sensor domain-containing protein**	NA	NA	**-7.02**	**1.39E-49**
**rcc01020**	**diguanylate cyclase/phosphodiesterase**	-3.04	5.45E-27	**-6.19**	**1.39E-42**
**rcc00567**	**nifA1; Nif-specific regulatory protein**	**-5.76**	**6.31E-28**	**-5.30**	**3.77E-12**
**rcc02629**	**diguanylate cyclase/phosphodiesterase**	NA	NA	**-5.00**	**2.49E-24**
**rcp00117**	**EAL domain-containing protein**	NA	NA	**-4.33**	**1.03E-35**
**rcc03176**	**PAS/PAC sensor domain-containing protein**	NA	NA	**-3.99**	**4.72E-55**
**rcc02198**	**histidine kinase**	NA	NA	**5.64**	**6.30E-06**
**rcp00066**	**NnrS family protein**	NA	NA	**6.81**	**3.90E-16**
**rcc01495**	**fusA2; translation elongation factor G**	2.69	2.25E-24	**7.00**	**1.44E-08**
**rcc02197**	**two component AraC family transcriptional regulator**	NA	NA	**7.71**	**1.04E-20**
**rcc01156**	**UspA domain-containing protein**	3.70	1.59E-09	**14.15**	**2.22E-52**
COG U: Intracellular trafficking, secretion, and vesicular transport					
**rcc02066**	**hemolysin D**	NA	NA	**-8.85**	**2.02E-26**
COG V: Defense mechanisms					
**rcc02065**	**secretion ATP-binding protein, HlyB family**	NA	NA	**-18.81**	**1.20E-139**
**rcc00886**	**ABC transporter ATP-binding protein**	2.93	2.01E-07	**6.19**	**2.53E-14**
**rcc01440**	**ABC transporter ATP-binding/permease**	**8.16**	**1.33E-73**	NA	NA

Genes highlighted in red indicate repression by RegA, genes highlighted in green indicate activation by RegA.
NA denotes No Value Avaluble as there was no significant change in expression observed in these conditions.

Table S4: Overlap between *Rba. capsulatus* RegA and *Rba. sphaeroides* PrrA regulons

RNA-seq overlap

R. sphaeroides Gene	R. capsulatus Gene	Annotation
RSP_0276	RCAP_rcc00028	idi1
RSP_1518	RCAP_rcc00045	regA1
RSP_0820	RCAP_rcc00436	cytB
RSP_1574	RCAP_rcc00436	cytB
RSP_1760	RCAP_rcc00543	hypothetical protein
RSP_2950	RCAP_rcc00550	lysR transcription factor
RSP_3306	RCAP_rcc00655	hypothetical protein
RSP_0274	RCAP_rcc00676	bchD
RSP_0273	RCAP_rcc00677	bchI
RSP_0272	RCAP_rcc00678	crtA
RSP_0269	RCAP_rcc00681	tspO
RSP_0265	RCAP_rcc00684	crtE
RSP_0260	RCAP_rcc00689	bchZ
RSP_0259	RCAP_rcc00690	pufQ
RSP_0257	RCAP_rcc00693	pufL
RSP_0256	RCAP_rcc00694	pufM
RSP_0254	RCAP_rcc00696	dxs1
RSP_0680	RCAP_rcc01172	hemE
RSP_0679	RCAP_rcc01173	hemC
RSP_2984	RCAP_rcc01447	hemA
RSP_2888	RCAP_rcc01722	BadM/Rrf2 family transcriptional regulator
RSP_2395	RCAP_rcc01723	ccpA
RSP_2864	RCAP_rcc01828	rpe1
RSP_3271	RCAP_rcc01829	cbbM
RSP_3268	RCAP_rcc01832	tkt1
RSP_3267	RCAP_rcc01833	cbbP
RSP_3266	RCAP_rcc01834	fbp
RSP_1467	RCAP_rcc02390	alkane 1-monooxygenase
RSP_2656	RCAP_rcc02430	nahG
RSP_2718	RCAP_rcc02462	hypothetical protein
RSP_0315	RCAP_rcc02532	pucC2
RSP_2386	RCAP_rcc02664	mtnA
RSP_1837	RCAP_rcc03091	mtnP
RSP_1836	RCAP_rcc03092	apt
RSP_1507	RCAP_rcc00282	aldehyde dehydrogenase
RSP_1012	RCAP_rcc00413	gabD
RSP_1880	RCAP_rcc00642	peptidoglycan binding domain-containing protein
RSP_0696	RCAP_rcc01157	ccoN
RSP_0694	RCAP_rcc01159	ccoQ

Similar ChIP peak locations

Peak	Start	End	Genes	Annotation
1	1	813	dnaA	chromosomal replication initiator protein
2	191712	193420	rcc_00165	NAD-dependent epimerase/dehydratase
3	295561	297348	rcc_00258	putative lipoprotein
			rcc_00259	porin family protein
4	756419	765975	crtF	carotenoid biosynthesis
			pufQ	cytochrome

			pufB	light harvesting complex
			pufA	light harvesting complex
			pufL	reaction center
			pufM	reaction center
5	905561	906752	ddl	D-alanine-D-alanine ligase
6	1564402	1565565	hemA	5-aminolevulinate synthase
7	2336371	2337762	rne	ribonuclease E
8	2719251	2722948	pucB	light harvesting complex
			pucA	light harvesting complex
			pucC2	light harvesting complex
			pucDE	light harvesting complex
9	2981202	2982457	rcc_02790	carD family transcriptional regulator

Table S4: Overlap between DEGsand ChIP peaks in PrrA (*Rba. sphaeroides*) and RegA (*Rba. capsulatus*) under anaerobic growth in minimal medium. Genes listed above in black type indicate regulation ≥ 2 fold in both species, genes listed above in red indicate regulation ≥1.5 fold in *Rba. capsulatus* and ≥ 2 fold in *Rba. sphaeroides*. Chip peaks shown are Rba. capsulatus peaks that were also found in *Rba. sphaeroides* upstream of similar genes.
